# Oxo dicopper anchored on carbon nitride for selective oxidation of methane

**DOI:** 10.1038/s41467-022-28987-1

**Published:** 2022-03-16

**Authors:** Pengfei Xie, Jing Ding, Zihao Yao, Tiancheng Pu, Peng Zhang, Zhennan Huang, Canhui Wang, Junlei Zhang, Noah Zecher-Freeman, Han Zong, Dashui Yuan, Shengwei Deng, Reza Shahbazian-Yassar, Chao Wang

**Affiliations:** 1 Department of Chemical and Biomolecular Engineering, Johns Hopkins University, Baltimore, MD 21218 USA; 2 College of Chemical and Biological Engineering, Zhejiang University, Hangzhou, 310027 China; 3 State Key Laboratory of Materials-Oriented Chemical Engineering, College of Chemical Engineering, Nanjing Tech University, Nanjing, 211816 P.R. China; 4 Institute of Industrial Catalysis, College of Chemical Engineering, Zhejiang University of Technology, Hangzhou, 310014 China; 5 School of Materials Science and Engineering, Zhengzhou University, Zhengzhou, 450001 China; 6 Department of Mechanical and Industrial Engineering, University of Illinois, Chicago, IL 60607 USA

**Keywords:** Heterogeneous catalysis, Chemical engineering, Catalytic mechanisms

## Abstract

Selective conversion of methane (CH_4_) into value-added chemicals represents a grand challenge for the efficient utilization of rising hydrocarbon sources. We report here dimeric copper centers supported on graphitic carbon nitride (denoted as Cu_2_@C_3_N_4_) as advanced catalysts for CH_4_ partial oxidation. The copper-dimer catalysts demonstrate high selectivity for partial oxidation of methane under both thermo- and photocatalytic reaction conditions, with hydrogen peroxide (H_2_O_2_) and oxygen (O_2_) being used as the oxidizer, respectively. In particular, the photocatalytic oxidation of CH_4_ with O_2_ achieves >10% conversion, and >98% selectivity toward methyl oxygenates and a mass-specific activity of 1399.3 mmol g Cu^−1^h^−1^. Mechanistic studies reveal that the high reactivity of Cu_2_@C_3_N_4_ can be ascribed to symphonic mechanisms among the bridging oxygen, the two copper sites and the semiconducting C_3_N_4_ substrate, which do not only facilitate the heterolytic scission of C-H bond, but also promotes H_2_O_2_ and O_2_ activation in thermo- and photocatalysis, respectively.

## Abstract (web summary)

Selective conversion of methane into value-added chemicals is a promising approach for utilization of hydrocarbon sources. Here the authors develop dimeric copper centers supported on graphitic carbon nitride (denoted as Cu_2_@C_3_N_4_) with >10% conversion and >98% selectivity toward methyl oxygenates in both thermo- and photo- catalytic reactions.

## Introduction

Selective conversion of methane to liquid hydrocarbons represents a promising approach toward efficient utilization of natural gas^1^. The present industrial route for such conversions relies on a two-step process by first reforming methane to generate synthesis gas (CO and H_2_) at elevated temperatures (>500 °C), and then reacting CO with H_2_ to form methanol or other liquid products^2,3^. However, this process is energy-intensive and economically nonviable for distributed sources such as flare gas^4^. More robust technologies toward direct conversion of methane into condensed energy carriers are thus demanded to facilitate transportation and storage^5^.

Direct, partial oxidation of methane to methyl oxygenates has received intensive attention in recent years^6,7^. The studies on early days use transition copper exchanged zeolites to catalyze the reaction between CH_4_ and O_2_, and employ a two-step chemical looping process to subsequentially activate O_2_ and desorb the products^8–10^. Despite the achievement of high selectivities, these reactions are still suffering from the low CH_4_ conversions (typically < 0.03%) and reaction rates (<30 μmol g_cata_^−1^ h^−1^)^11–13^. Later on, partial oxidation of methane in a single step has been demonstrated by using non-O_2_ oxidizers such as oleum^14^), selenic acid^15^) and H_2_O_2_^16–18^, but the cost associated with these oxidizing agents holds back their practical implementations^19^. More recent efforts have thus turned into in-situ generation of H_2_O_2_ from O_2_ by using selective oxygen reduction catalysts such as Au-Pd containing zeolites^20^. Alternatively, photoexcitation using visible light is proposed to be advantageous with near-room temperature activation of CH_4_, mitigating the concern of over oxidation to form CO_2_ upon heating^21,22^. But the reported photocatalytic oxidation of methane is still limited by relatively low methane conversions (<1%) and productivities (0.001~150 mmol g_cata_^−1^ h^−1^)^22^, as the commonly used photocatalysts have quite large bandgaps (e.g., ~3.2 eV for TiO_2_^23^ and ~3.4 eV for ZnO)^21^ and may only activate methane via the Fenton or homolytic mechanisms that have relatively sluggish kinetics^24^. In this aspect, graphitic carbon nitride (g-C_3_N_4_) represents a promising photocatalytic substrate with a modest band gap in the range of 2.7-2.9 eV^25–27^. Its abundant nitrogen sites have been shown in many reports to be capable of anchor atomically dispersed transition metal sites^28^. It thus becomes interesting to investigate the potential coordination of active Cu sites on g-C_3_N_4_ and examine their synergies in partial oxidation of methane.

**Fig. 1:**
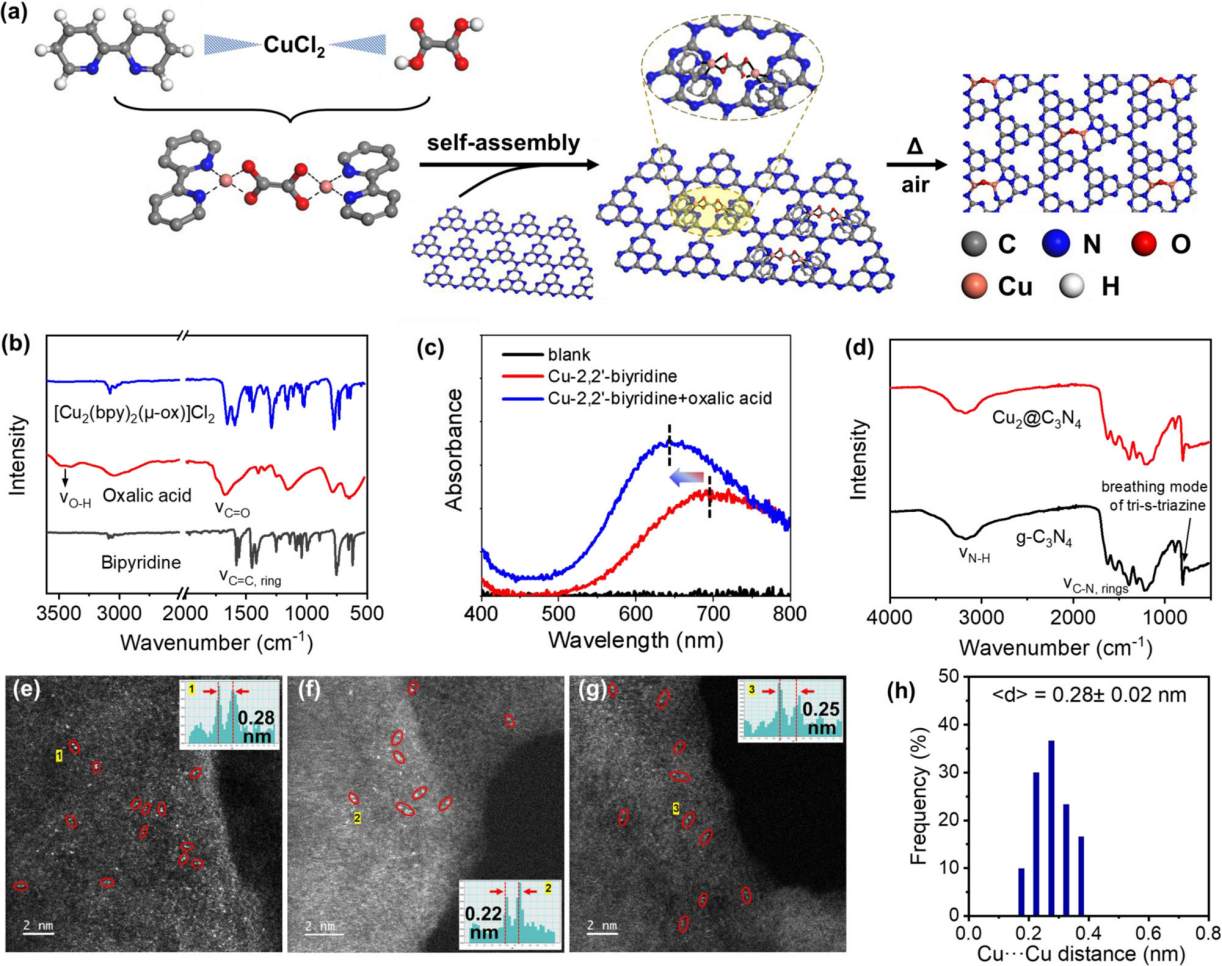
Synthesis of the Cu_2_@C_3_N_4_ catalysts. **a** Scheme illustration of synthetic route. **b**, **c** Characterization of the Cu-dimer precursor, [Cu_2_(bpy)_2_(μ-ox)]Cl_2_ complex, using FTIR (**b**) and UV-vis DRS (**c**). **d** Comparison of FTIR spectra for Cu_2_@C_3_N_4_ and g-C_3_N_4_. **e**–**g** Representative HAADF-STEM images of Cu_2_@C_3_N_4_, with the insets showing line-scanning intensity profiles of Cu dimers. **h** Statistical distribution of the Cu-Cu distance in the Cu dimers derived from the STEM images.

Here we report on Cu_2_@C_3_N_4_ as highly efficient catalysts for partial oxidation of methane. The dimeric copper catalysts were synthesized by supporting an (oxalato)(bipyridine)copper(II) complex, [Cu_2_(bpy)_2_(*μ*-ox)]Cl_2_, on g-C_3_N_4_ and then applying a mild thermal treatment in air (Fig. 1a). The derived catalysts contained dicopper-oxo centers anchoring on g-C_3_N_4_ via four Cu-N bonds (two for each copper atom), as characterized by using STEM, XPS and XAS, and also confirmed with atomistic simulations. The obtained copper-dimer catalysts were first evaluated for thermal oxidation of methane using H_2_O_2_ as the oxidizer, and then further applied for photocatalytic oxidation of methane with O_2_. Mechanisms governing the observed catalytic enhancements toward selective oxidation of methane were interpreted via combining computational simulation of the reaction pathways, spin-trapping EPR analysis of possible radical intermediates, and in situ XPS measurements under light irradiation.

## Results and discussion

### Synthesis and characterization of Cu_2_@C_3_N_4_

The copper-dimer precursor [Cu_2_(bpy)_2_(μ-ox)]Cl_2_ was first prepared by a complexation reaction of copper chloride (CuCl_2_), 2,2,-bipyridine and oxalic acid^29^. The g-C_3_N_4_ substrate was grown by calcination of urea at 550 °C^30^. Cu_2_@C_3_N_4_ catalysts were synthesized by self-assembly of the dimeric copper complex on g-C_3_N_4_^31^ and then treating the mixture in air at 250 °C to immobilize the copper species (Fig. 1a). The loading of Cu was determined to be 0.35 wt% by using inductively coupled plasma mass spectrometry (ICP-MS).

The complexation of pyridine, Cu^2+^ and oxalate (C_2_O_4_^2−^) to form an organometallic compound was confirmed by using Fourier transform infrared spectroscopy (FTIR). The hydroxyl (O-H) and carbonyl (C = O) stretching features around 3450 and 1670 cm^−1^, respectively, associated with oxalic acid disappeared after the reaction. This was accompanied with the blue shift of the characteristic band (attributed to the asymmetric stretching of the pyridyl ring) of 2,2′-pyridine at ca. 1580 cm^−1^ to ca. 1650 cm^−1^, a consequence of its chelation with Cu^2+^ (Fig. 1b)^32^. Correspondingly, the *d-d* transition of Cu^2+^ at 650–700 nm had a blue shift of 48 nm in the ultraviolet-visible diffuse reflectance spectroscopy (UV-vis DRS) patterns (Fig. 1c). The FTIR spectra of g-C_3_N_4_ and Cu_2_@C_3_N_4_ exhibited the stretching vibration modes characteristic of the −NH group around 3180 cm^−1^, C-N heterocycles in the wavelength range of 1100–1650 cm^−1^ and the breathing mode of tri-*s*-triazine units at 810 cm^−1^ (Fig. 1d). Comparing to [Cu_2_(bpy)_2_(μ-ox)]Cl_2_, Cu_2_@C_3_N_4_ presented no more infrared features associated with the dimeric copper complex, indicating the complete removal of organic ligands during the immobilization process. This was further confirmed with thermogravimetric analysis (TGA) (Supplementary Fig. 1). The [Cu_2_(bpy)_2_(μ-ox)]Cl_2_/g-C_3_N_4_ mixture lost ~6% of its initial weight upon annealing in air at up to 250 °C, which is close to the expectation estimated based on the ligand content (~80 wt%) of [Cu_2_(bpy)_2_(μ-ox)]Cl_2_ and its ratio relative to the carbon nitride substrate (~8%) used in the synthesis. X-ray diffraction (XRD) patterns collected for the Cu_2_@C_3_N_4_ catalysts only show the (001) and (002) peaks associated with g-C_3_N_4_, with the absence of copper metal or oxide features indicating the highly dispersed nature of copper species (Supplementary Fig. 2). Atomic structures of the dimeric copper moieties were resolved by using aberration correction high angle annular dark field scanning transmission electron microscopy (HAADF-STEM) imaging (Fig. 1e-g). The collected STEM images exhibit a large number of adjacent, paired bright dots (labeled with red circles, <0.35 nm in size for each dot) distributed on a substrate of lower contrasts. These small bright dots can be attributed to atomically dispersed Cu considering their much higher Z contrast (M = 65 for Cu) than C_3_N_4_ (M = 12 or 14). Line-profile scanning for ~100 pairs of such bright dots give an average distance of 2.8 (±0.2 Å) (Fig. 1h). This is much shorter than the value (5.2 Å) for the two copper atoms within [Cu_2_(bpy)_2_(μ-ox)]Cl_2_, again confirming the reconstruction and condensation of the copper-dimer moieties as a result of the removal of organic ligands in the synthesis.

**Fig. 2:**
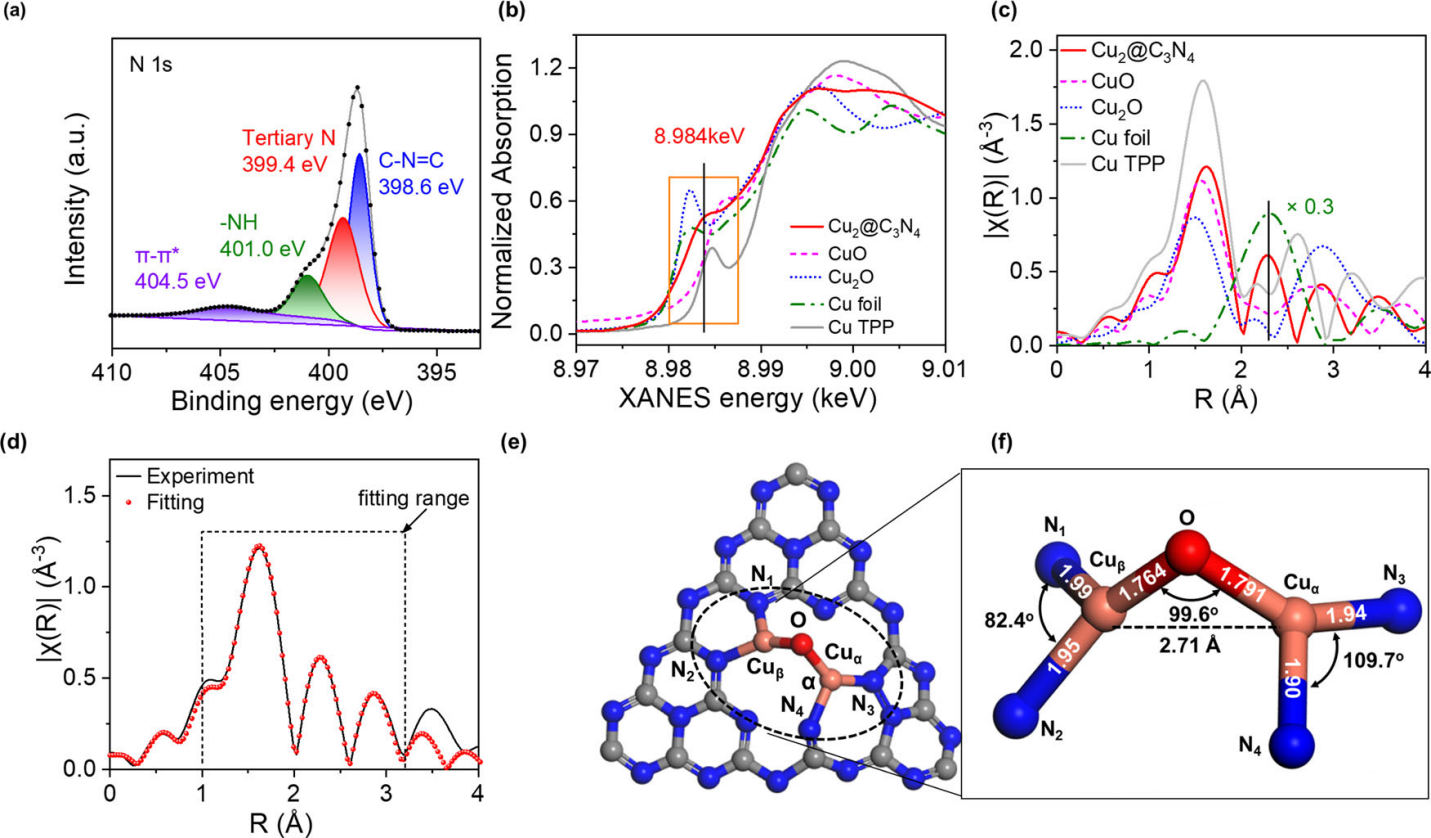
Characterization of the Cu_2_@C_3_N_4_ catalysts. **a** XPS spectrum at the N *1* *s* edge and the corresponding deconvolution. **b** XANES spectra and (**c**) k^2^-weighted EXAFS spectra at the Cu K edge, with Cu foil, Cu_2_O, CuO, and Cu-TPP (one Cu coordinated with for N atoms) as the reference. **d** Fitting of the EXAFS spectrum with consideration of both monomeric and dimeric Cu sites. **e** The simulated s**t**ructure model of dicopper-oxo center. **f** Geometric parameters of the dicopper-oxo center determined for Cu_2_@C_3_N_4_.

Chemical nature of the Cu dimers in Cu_2_@C_3_N_4_ was probed by using X-ray photoelectron spectroscopy (XPS) and X-ray absorption spectroscopy (XAS). The N *1* *s* XPS spectrum exhibits a broad feature with the binding energy ranging from 397 to 408 eV (Fig. 2a). This feature can be deconvoluted into four peaks centered at 398.6, 399.4, 401.0, and 404.5 eV, which can be assigned to pyridinic (C − N = C), tertiary (−N<), pyrrolic (−NH) nitrogen, and π-π* transition of C = N or uncondensed terminal amine groups in g-C_3_N_4_, respectively^33,34^. The Cu *2p* spectrum shows two peaks at 932.5 eV and 952.3 eV, which are characteristic of Cu(I) or Cu^0^ (Supplementary Fig. 3)^35^. However, the XPS analysis (as well as the corresponding Auger electron spectrum, AES) was unable to explicitly determine the oxidation state of Cu due to the reduced signal-to-noise ratio associated with the low copper content in the catalysts. The copper oxidation state in Cu_2_@C_3_N_4_ was better resolved by using X-ray Absorption Near Edge Spectroscopy (XANES) (Fig. 2b). The Cu K edge spectrum exhibits a pre-edge transition at 8984 eV, which falls between the peaks associated with Cu_2_O (8983 eV) and CuO (8986 eV). This indicates an intermediate oxidation state between +1 and +2 for Cu in Cu_2_@C_3_N_4_. Noticeably, our results do not support the picture with a mixture of Cu(I) and Cu(II), as the *1* *s* → *3d* transition at 8977 eV, a feature characteristic of Cu^2+^ (as shown for the references CuO and copper tetraphenylporphyrin (Cu-TPP) in Supplementary Fig. 4)^35^, is absent in the spectrum of Cu_2_@C_3_N_4_. The partial oxidation state (between +1 and +2) of Cu within the copper dimers supported on g-C_3_N_4_ can be viewed as a result of the semiconducting nature of the substrate. This is distinguished from the case for extensively studied copper exchanged zeolites, in which the dicopper-oxo centers ([Cu-O-Cu]^2+^) anchor on the Al sites with localized negative charges and have an oxidation state of +2 for both Cu atoms^9,36,37^.

The atomic structure of the Cu dimers was resolved by combining extended X-ray absorption fine structure (EXAFS) analysis and atomistic modeling based on DFT calculations. Figure 2c compares the k2-weighted Cu K edge EXAFS spectra for Cu_2_@C_3_N_4_, Cu foil, Cu_2_O, CuO, and Cu-TPP (with single-atom Cu^2+^ coordinating to four pyrrolic N, Supplementary Fig. 4). The Cu_2_@C_3_N_4_ catalyst exhibits first-shell scattering at 1.62 Å in R space (prior to phase correction), which is proximate to the values, 1.59 and 1.55 Å, found for Cu-TPP and CuO, respectively. This is distinct from the cases for Cu_2_O and Cu, the first-shell scattering of which locates at 1.50 and 2.30 Å, respectively. From these observations, we tentatively assign the primary scattering pair at 1.62 Å in the R-space spectrum of Cu_2_@C_3_N_4_ to be Cu-N or Cu-O bonding. To fit the EXAFS spectrum, a total of 9 possible Cu-dimer configurations was postulated, based on which DFT calculations were performed to relax the structures and determine bonding distances and angles (Supplementary Fig. 5 and Table 1). Considering the presence of minor Cu monomers observed in the STEM images (Fig. 1e–g), we have applied a linear combination of Cu monomers and dimers to fit the EXAFS spectrum. Various possible Cu-dimer configurations have been considered, with the corresponding EXAFS spectra compared to the experimental results to identify the best fit (Fig. 2d, e; also see Supplementary Figs. 6–7). It is estimated that 72.4% of the Cu atoms are in the dimeric configuration, close to the value (~70%) derived from statistical analysis of the STEM images. The determined copper-dimer structure comprises two Cu atoms bridged by an O atom, with the Cu-O bonds having lengths of 1.76 Å and 1.79 Å and an included angle (∠Cu-O-Cu) of 99.6^o^ (Fig. 2f, Table 1). Each Cu atom is coordinated to two N atoms on the C_3_N_4_ framework, with the bonding distance varying from 1.90 to 1.99 Å and the bonding angle (∠N-Cu-N) being 82^o^ for Cu_α_ and 110^o^ for Cu_β_ (Fig. 2f). Noticeably, the identified configuration with best fitting to the EXAFS spectrum also has the lowest (most negative) formation energy among the various configurations, in line with the expectation for stable atomic structures in the real catalysts (Supplementary Table 1). Our combined EXAFS analysis and DFT calculations resolved the Cu-Cu distance in the Cu dimers to be ~2.71 Å (Table 1), which is in agreement with the average Cu-Cu distance measured from the STEM images (Fig. 1h). It is noted that this value is much smaller than the Cu-Cu distance (4.10 Å) associated with the dicopper-oxo center ([Cu-O-Cu]^2+^) in Cu-ZSM-5^37^. Furthermore, Bader charge analysis based on DFT calculation shows that the Cu atoms in the Cu dimers have oxidation states of +1.63 and +1.72 (Supplementary Fig. 7), resembling the results derived from XANES spectra (see the above discussion for Fig. 2b).

**Table 1:** Structural parameters being used to fit the EXAFS spectrum for Cu_2_@C_3_N_4_ with consideration of both monomeric and dimeric Cu sites.

Scattering path	CN	Distance (Å)	σ2(Å^2^)	R-factor
Cu-O	1.25 ± 0.20	1.77 ± 0.01	0.0056 ± 0.0006	0.009 ± 0.001
Cu-N (Cu dimer)	1.84 ± 0.29	1.99 ± 0.01		
Cu-N (Cu monomer)	2.42 ± 0.32	1.98 ± 0.01		
Cu-Cu	0.88 ± 0.15	2.71 ± 0.01	0.0038 ± 0.0004	

From the above discussion, we can see that the dimeric copper centers in Cu_2_@C_3_N_4_ have distinct atomic structures and electronic chemical properties form their counterparts confined in zeolites. We hypothesize that their non-integer oxidation state (intermediate between +1 and +2) and reduced cluster size (smaller Cu-Cu distance as compared to Cu-ZSM-5) would lend them exquisite catalytic performance for selective oxidation of methane^36^.

### Thermocatalytic oxidation of methane with H_2_O_2_

**Fig. 3:**
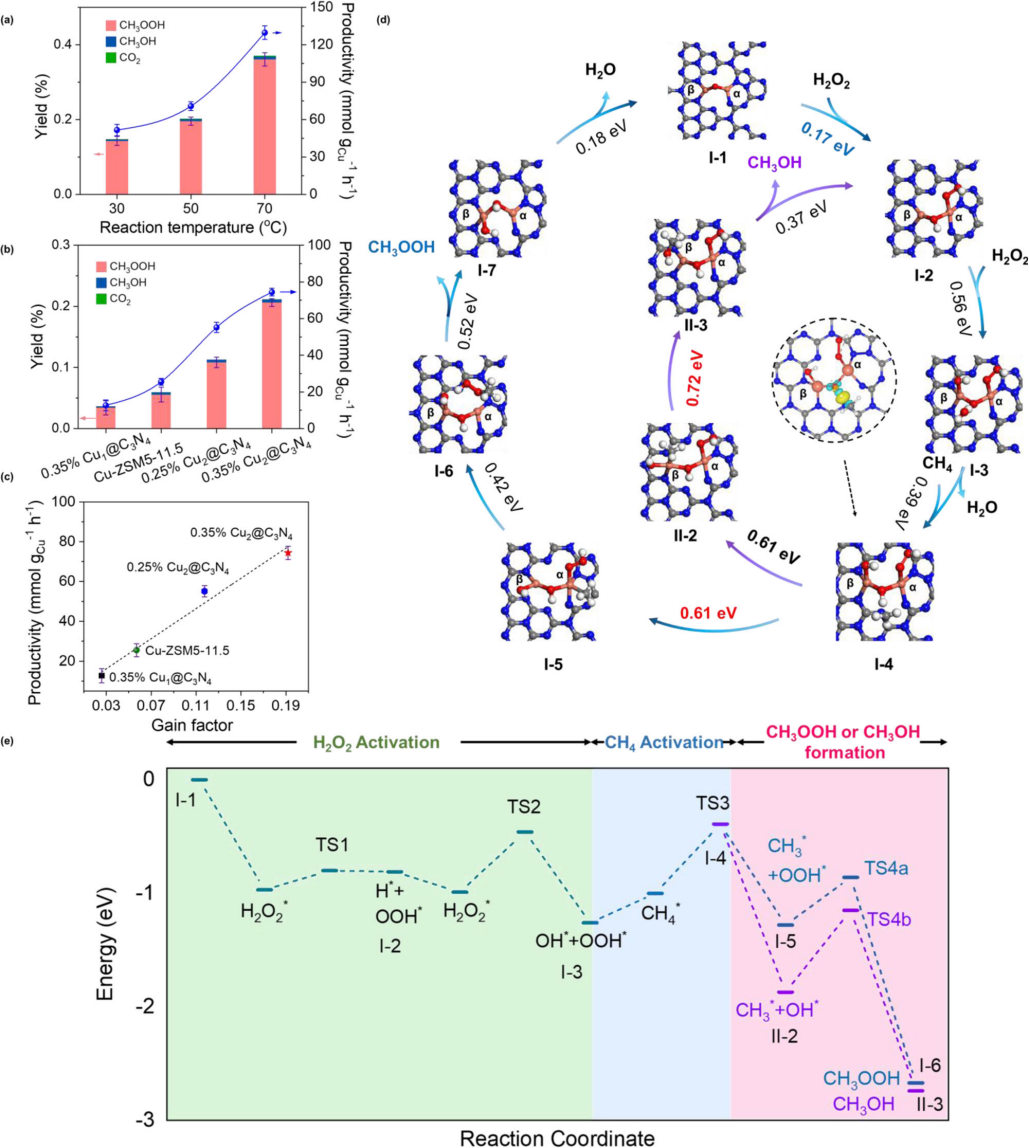
Thermo-catalytic oxidation of CH_4_ with H_2_O_2_. **a** Yields and productivity of methyl oxygenates at different reaction temperatures. **b** Comparisons of product yields and productivity over different catalysts. **c** Correlation between productivity of methyl oxygenates and gain factor for different catalysts. **d** Simulated pathways for the reaction between CH_4_ with H_2_O_2_ on the Cu_2_@C_3_N_4_ catalysts, with the middle inset illustrating the electron distribution of the CH_4_ molecule being activated on the bridging oxygen site. Energy barriers are also given for the associated molecular transformations. **e** The DFT calculated free energy diagram for the Cu_2_@C_3_N_4_-catalyzed partial oxidation of CH_4_ with H_2_O_2_. Three stages consisting of H_2_O_2_ activation, CH_4_ activation and methyl oxygenates formation are distinguished with different colors. The error bars presented in (**a**–**c**) indicate the statistical distribution derived from three independent measurements.

The Cu_2_@C_3_N_4_ catalysts were first evaluated for the thermocatalytic oxidation of CH_4_ (Supplementary Figs. 8–12). This was conducted using a continuous stirred-tank reactor (CSTR) filled with 0.2 mM of H_2_O_2_ and 0.1 MPa of CH_4_ (see Experimental Methods in the Supplementary Materials). Methyl oxygenates (CH_3_OH and CH_3_OOH) were found to be the primary products, with the yield achieving 0.14% within 30 min of reaction at 30 °C (Fig. 3a). As previously reported, the generated CH_3_OOH can be facilely reduced to CH_3_OH under ambient conditions (Supplementary Fig. 12)^19,38^. The yield of methyl oxygenates increased to 0.37% at 70 °C, corresponding to the increase of productivity from 51.6 to 129.7 mmol g_Cu_^−1^ h^−1^. Albeit the increase of reaction rate, the rise of reaction temperature is accompanied with the increase of CO_2_ selectivity from 0.8% at 30 °C to 5.0% at 70 °C (Supplementary Fig. 13). Similarly, elongated operations also led to the yield of more CO_2_ (Supplementary Fig. 13). The cyclability tests showed that the Cu_2_@C_3_N_4_ catalyst was stable throughout the methane oxidation reaction with H_2_O_2_. In five consecutive measurements by refilling CH_4_ and H_2_O_2_, the Cu_2_@C_3_N_4_ catalyst exhibited indiscernible change in reactivity and product distribution, with the productivity of methyl oxygenates found to be consistent at ~70 mmol g_Cu_^−1^ h^−1^ at 50 °C (Supplementary Fig. 14). Furthermore, the atomic structure of dicopper-oxo centers was confirmed to remain intact after reaction by performing HADDF-STEM imaging and EXAFS analysis on the spent Cu_2_@C_3_N_4_ catalyst after 6 h of reaction (Supplementary Fig. 15 and Supplementary Table 2).

Considering that the bare g-C_3_N_4_ substrate is inactive for CH_4_ oxidation (Supplementary Fig. 16), the dicopper-oxo centers can be identified as the active sites in Cu_2_@C_3_N_4_. Considering that monomeric Cu in copper exchanged zeolites^10,39,40^ or metal−organic frameworks^41^ has also been discussed to be active for CH_4_ oxidation, we have performed comparative studies on a single-atom control (Cu_1_@C_3_N_4_) using the same g-C_3_N_4_ substrate. This catalyst was prepared by using a vapor-migration strategy^42^ with the Cu loading controlled to be also at ~0.35 wt%, with the single-atom dispersion confirmed by using XAS (Supplementary Fig. 17). Catalytic studies showed that Cu_1_@C_3_N_4_ was merely active for methane oxidation, delivering a yield of only 0.03% (versus 0.2% by Cu_2_@C_3_N_4_) for methyl oxygenates at 50 °C (Fig. 3b). The low reactivity of Cu_1_@C_3_N_4_ indicates that Cu monomers, if present in the Cu_2_@C_3_N_4_ catalysts, would not make significant contributions to the observed high methane partial oxidation activity, and also underlines the necessity of having dicopper-oxo centers for catalyzing the partial oxidation of methane. The activity of Cu_2_@C_3_N_4_ is also substantially enhanced as compared to copper exchanged zeolites. A comparative study of Cu-ZSM5 with a Si/Al ratio of 11.5 and full exchange (Cu/Al ~ 0.51) using similar reaction conditions only delivered a productivity of 25.5 mmol g_Cu_^−1^ h^−1^ for methyl oxygenates at 50 °C, as compared to 74.4 mmol g_Cu_^−1^ h^−1^ for Cu_2_@C_3_N_4_ at this temperature (Fig. 3b). Note that the copper species in this Cu-ZSM-5 catalyst is also predominantly present in the form of dicopper-oxo centers^36^, similar to that identified in Cu_2_@C_3_N_4_ (as shown in Fig. 2f). These results thus indicate that the dimeric Cu supported on carbon nitride is much more reactive for the oxidation of methane with H_2_O_2_ than their counterparts confined in zeolites.

In the partial oxidation of methane with H_2_O_2_, the efficiency of utilizing the peroxide oxidizer (instead of producing O_2_ through a disproportionation reaction) is an important metric for evaluating the performance of catalysts^19,43^. This is usually assessed by comparing the “gain factor” that is defined as the molar ratio between the produced methyl oxygenates (CH_3_OH and CH_3_OOH) and the consumed H_2_O_2_^19^. Post-reaction titration of the concentration of residual hydrogen peroxide using cerium sulfate^44^ (Supplementary Fig. 18) showed that the Cu_2_@C_3_N_4_ catalyst had a gain factor of 0.19 (Fig. 3c and Supplementary Fig. 19). In comparison, the gain factor was determined to be only 0.03 and 0.06 for Cu_1_@C_3_N_4_ and Cu-ZSM5-11.5, respectively. It is interesting that the gain factor exhibited dependence on the copper loading in the dimer catalyst. A Cu_2_@C_3_N_4_ catalyst of reduced loading (0.25 wt%) had a gain factor of 0.12, which is lower than that for the normal catalyst with 0.35 wt% of copper. Moreover, correlation between the productivity of methyl oxygenates and the gain factor gives rise to a linear relationship, underscoring its meaning of describing the reactivity between methane and H_2_O_2_ on a given catalyst (Fig. 3c)^19,45^.

To understand the enhanced reactivity of Cu_2_@C_3_N_4_ for methane partial oxidation, we have performed DFT calculations to simulate the reaction pathways on the dicopper-oxo centers (Figs. 3d, e; also see Supplementary Fig. 20 and Supplementary Table 3). It is predicted that the reaction starts with sequential activation of two H_2_O_2_ molecules on the copper-dimer centers through radical mechanisms^24,46,47^. The first hydrogen peroxide molecule is dissociated via H_2_O_2_ → ‧OOH + *H, where the hydrogen adsorbs on the bridging oxygen and the ‧OOH radical migrates onto Cu_α_ to become a peroxyl (*OOH) adsorbate. The second hydrogen peroxide undergoes H_2_O_2_ → ‧OH + *OH with the hydroxyl group adsorbing on Cu_β_ and the ‧OH radical recombines with the *H on the bridging oxygen site to form a H_2_O molecule. The involvement of ‧OOH and ‧OH in H_2_O_2_ activation was corroborated by the observation of these radicals in the electron paramagnetic resonance (EPR) spectroscopic studies by using 5,5′-Dimethyl-1-pyrroline-N-oxide (DMPO) as the radical trap (Supplementary Fig. 21)^48^. The generation of radicals is the rate limiting factor in both cases of H_2_O_2_ activation, which is predicted to have a kinetic barrier of 0.17 (for ‧OOH) or 0.56 (for ‧OH) eV. Noticeably, these barriers are substantially lower than the corresponding values found for the single-atom Cu sites (1.3 and 1.5 eV, Supplementary Fig. 22) and the dicopper-oxo centers confined in zeolites (0.58 and 0.81 eV in Cu-ZSM-5)^7,49^, in line with the higher gain factor and enhanced utilization of H_2_O_2_ as observed on the Cu_2_@C_3_N_4_ catalysts (Fig. 3c). The enhanced H_2_O_2_ activation on Cu_2_@C_3_N_4_ could be ascribed to the π-conjugated heterocyclic rings and the semiconducting nature of the C_3_N_4_ substrate, which is known for accommodation of charge transfer and able to supply electrons to the dicopper-oxo center for stabilization of the oxygenated adsorbates^50–53^. The C_3_N_4_ supported Cu dimers are thus believed to be more advantageous than their zeolitic counterparts for catalyzing the redox chemistries being examined here.

Following the activation of H_2_O_2_, methane is introduced to the dicopper-oxo center with one of the C-H bond attacked by the bridging oxygen (Fig. 3d, e). This C-H bond dissociation has a modest energy barrier of 0.61 eV (vs. ~0.71 eV in the case of Cu-ZSM-5)^10,39,40^. While the generated H adsorbs on the bridging oxygen, the methyl group migrates on to the adjacent Cu sites. Hereby the C-H bond dissociation is believed to be *heterolytic* instead of *homolytic* or the *Fenton* type, as no ‧CH_3_ radicals were observed using EPR (Supplementary Fig. 23)^19,24,38^. The heterolytic dissociation of C-H bond is believed to be essential for partial oxidation of methane at high selectivities, as the other two activation mechanisms via ‧CH_3_ radicals are typically accompanied with over oxidation to form substantial amounts of CO_2_. Compared to the case in Cu-ZSM-5^37^, the Cu dimers supported on g-C_3_N_4_ have shorter Cu-O bond length (1.77 Å vs 1.88 Å) and smaller∠Cu-O-Cu (99.6° vs 135°), which are believed to sterically favor the heterolytic cleavage of the C-H bond and facilitate the transer of the -CH_3_ group. Noticeably, the -CH_3_ group can adsorb on either Cu_α_ or Cu_β_, where the reaction bifurcates into two possible pathways. On the one hand, *CH_3_ on Cu_α_ recombine with the *OOH on this site to form *CH_3_OOH. On the other hand, it can also recombine with *OH on Cu_β_ to form *CH_3_OH. Desorption of these adsorbates gives rise to the corresponding methyl oxygenates. While the rate is limited by the *CH_3_ +*OH → *CH_3_OH recombination on Cu_β_ (with a barrier of 0.72 eV), the highest barrier for the CH_3_OOH pathway is found to be the desorption of *CH_3_OOH (0.52 eV). Overall, the CH_3_OOH pathway associated with Cu_α_ is energetically more favorable than the CH_3_OH pathway with Cu_β_, explaining the experimentally observed much higher yield of CH_3_OOH than CH_3_OH. The pathways as revealed in Fig. 3d emphasizes the synergy among the two Cu atoms and the bridging O in catalyzing the complex reaction involving multiple molecules (e.g., CH_4_ + 2H_2_O_2_ → CH_3_OOH + 2H_2_O), which is a unique feature of the carbon nitride supported dimeric copper centers. An analogous reaction mechanism was also proposed in the partial oxidation of methane with H_2_O_2_ catalyzed by Au-Pd colloids^19^.

### Photocatalytic oxidation of methane with O_2_

Despite the selective oxidation of methane obtained with Cu_2_@C_3_N_4_, the thermocatalytic reaction still relies on the use of H_2_O_2_ as oxidant, which is not a readily available feeding in industry. Moreover, the low CH_4_ conversions (<1%) also limits the potential of this process for practical implementations. Considering that g-C_3_N_4_ is a semiconductor (with a bandgap of 2.7–2.9 eV^25,26^) with demonstrated photocatalytic applications^27^, we have turned to photocatalysis to overcome the limitation of thermocatalytic reactions. Photocatalytic oxidation of methane was carried out at 50 °C by applying near-edge excitation (300 W Xenon lamp equipped with a 420 nm bandpass filter) and using O_2_ as the oxidant (Supplementary Fig. 24). It is hypothesized that photoexcitation can efficiently activate O_2_ and generate the oxygenates (*OOH and *OH), mimicking and improving the role that H_2_O_2_ played in the reaction^21,22,54^.

**Fig. 4:**
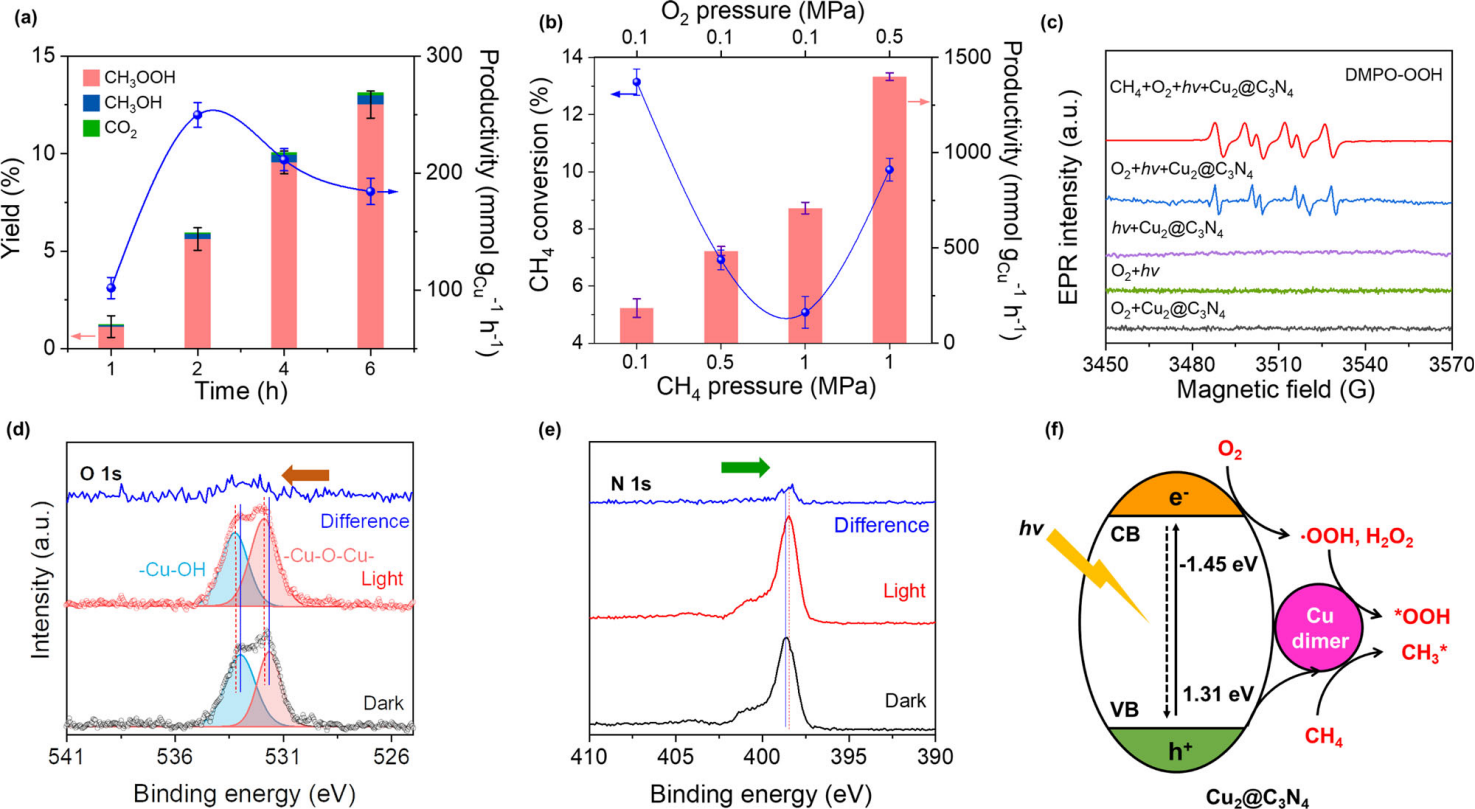
Photo-catalytic oxidation of CH_4_ with O_2_. **a** Yields and productivity of methyl oxygenates as a function of reaction time at 0.1 MPa CH_4_ and 0.1 MPa O_2_. **b** CH_4_ conversions and productivity of methyl oxygenates at different CH_4_ and O_2_ partial pressures. **c** EPR spectra recorded for various control experiments using DMPO as the radical trapping agent. **d**, **e** In situ irradiation XPS spectra collected at the O *1* *s* (**d**) and N *1* *s* (**e)** edges. **f** Schematic illustration of the photocatalytic oxidation of CH_4_ with O_2_ catalyzed by Cu_2_@C_3_N_4_. The values “−1.45 and 1.31 eV” label the estimated position of dicopper-oxo states in the band structure of g-C_3_N_4_, as determined by performing the UV-vis DRS and UPS spectra analysis of Cu_2_@C_3_N_4_. The error bars shown in (**a**, **b**) indicate the statistical distribution derived from three independent measurements.

The photocatalytic reaction gave much higher conversions of methane than the thermocatalytic process, reaching 1.3% at 1 h (Fig. 4a). The methane conversion increases with time, reaching ~13.1% at 6 h, where the products were found to be still dominated by CH_3_OOH and CH_3_OH (98.9% selectivity, Supplementary Fig. 25). The productivity of methyl oxygenates reached the peak value of 249.7 mmol g_Cu_^−1^ h^−1^ at 2 h, representing an improvement factor of ~3.6 as compared to the thermocatalytic reaction. Further improvement of the productivity was obtained by raising the partial pressure of methane (*P*_CH4_). As *P*_CH4_ increased from 0.1 to 1 MPa (at *P*_O2_ = 0.1 MPa, while the total pressure was kept constant at 3 MPa), the productivity escalated from 184.3 to 709.8 mmol g_Cu_^−1^ h^−1^, albeit with the methane conversion reducing from 13.1 to 5.1 (at 6 h, Fig. 4b and Supplementary Fig. 26). The improvement of productivity at higher *P*_CH4_ can be ascribed to the increased concentration of dissolved methane in the aqueous solution^20^. The low conversion of methane at high *P*_CH4_ was likely limited by the inadequacy of oxygen. At *P*_O2_ = 0.5 MPa and *P*_CH4_ = 1 MPa, a methane conversion of 10.1% was obtained with >98% selectivity toward methyl oxygenates, corresponding to an even higher productivity of 1399.3 mmol g_Cu_^−1^ h^−1^. A comprehensive comparison to the literature results under similar reaction conditions indicate that this represents the highest activity for partial oxidation of methane, with improvement factors of at least >10 (Supplementary Table 4).

The photocatalytic oxidation of methane with O_2_ was confirmed by conducting control experiments under various conditions (Supplementary Table 5). In particular, the Cu_2_@C_3_N_4_ catalyst was found to be inactive in darkness (while the other conditions were kept the same), ruling out the involvement of thermocatalytic reaction between CH_4_ and O_2_ in the photocatalytic studies. The photocatalytic activity of bare g-C_3_N_4_ was also nearly negligible, underlining the role of Cu dimers in catalyzing the related molecular transformations. The generation of active peroxide species in situ during the photocatalytic reaction was confirmed by performing EPR spectroscopic studies by also using DMPO as the radical trapping agent (Fig. 4c). The spectra recorded under visible light irradiation, in both cases with and without methane, show the fingerprints of ‧OOH radicals, which can be assigned to the spin of unpaired electrons on oxygen^21,45,54^. Similar to the findings from photocatalytic studies, such signals were not observed from the controls in the absence of O_2_, Cu_2_@C_3_N_4_, or light. These ‧OOH radicals are likely derived from the thermal activation of H_2_O_2_ (as observed in the thermocatalytic studies, Supplementary Fig. 21), which was produced from photocatalytic reduction of O_2_ in situ^55–57^. It thus becomes evident that Cu_2_@C_3_N_4_ is not only a good thermocatalyst for partial oxidation of methane with H_2_O_2_, but also an exceptional photocatalyst when the oxidant is replaced by O_2_.

In addition to the reduction of O_2_ to peroxides, the photon excitation is also believed to enhance the methane activation. This was revealed by using in situ irradiation X-ray photoelectron spectroscopy (ISI-XPS)^58^ to examine charge transfer between the dimeric copper center and the C_3_N_4_ substrate (see the Experimental Methods in the Supplementary Materials). As shown in Fig. 4d, the XPS spectra collected on hydrated Cu_2_@C_3_N_4_ in darkness exhibited two O *1* *s* peaks at ca. 533.0 and 531.7 eV, which can be assigned to the oxygen binding to Cu, i.e., -Cu-OH and -Cu-O-Cu-, respectively^59,60^. Under light irradiation (400~500 nm), both of these two peaks had a blue shift of ~0.5 eV. Similar observations were obtained at the Cu *2p* edge (Supplementary Fig. 27). Meanwhile, a red shift of the N *1* *s* peak associated with C_3_N_4_ was observed, from 398.8 eV in darkness to 398.2 eV under light irradiation (Fig. 4e). Such phenomena consistently point to the transfer of holes (rather than electrons) from the g-C_3_N_4_ substrate to the dicopper-oxo center, where CH_4_ is activated and oxidized to form *CH_3_. Meanwhile, the excited electrons in the g-C_3_N_4_ substrate lead to the reduction of O_2_ and formation of H_2_O_2_, which then migrates or diffuses onto the dicopper-oxo center and gets activated to form *OOH or *OH. In the following, these oxygen species recombine with *CH_3_ to form methyl oxygenates, as in the case of thermocatalytic reactions (Fig. 4f and Supplementary Figs. 28–29). Similar phenomena of charge transfer induced catalytic enhancements have previously been reported in photocatalysis using TiO_2_ based photocatalysts^61–63^.

In conclusion, we have developed new dimeric copper catalysts for partial oxidation of methane. These catalysts were synthesized by immobilization of a copper-dimer organometallic complex on graphitic carbon nitride, and dicopper-oxo centers were characterized to anchor on this substrate via Cu-N bonding. The derived Cu_2_@C_3_N_4_ catalysts were first examined for thermocatalytic oxidation of methane with H_2_O_2_, and then studied for photocatalytic reactions with O_2_ being used as the oxidant. Enhanced catalytic activities were demonstrated in both cases as compared to the other reported catalysts under similar reaction conditions, achieving improvement factors of more than an order of magnitude. Synergy of the bridging oxygen, the two copper sites, and the semiconducting C_3_N_4_ substrate has been revealed to promote H_2_O_2_ and O_2_ activation and the heterolytic scission of CH_4_. Our work highlights the great potential of carbon nitride supported dimeric copper centers in catalyzing redox chemical reactions.

## Methods

### Materials and Chemicals

The following chemicals were purchased and used as-received without further purification: Copper(II) chloride dihydrate (CuCl_2_·2H_2_O, ACS grade, Sigma Aldrich), 2,2,-bipyridine (C_10_H_8_N_2_, reagent grade, Sigma Aldrich), oxalic acid (HO_2_CCO_2_H, reagent grade, Alfa Aesar), urea (NH_2_CONH_2_, ACS grade, Sigma Aldrich), dicyandiamide (NH_2_C( = NH)NHCN, ACS grade, Sigma Aldrich), copper(II) acetylacetonate (Cu(acac)_2_, ACS reagent, Sigma Aldrich), oleylamine (CH_3_(CH_2_)_7_CH = CH(CH_2_)_8_NH_2_, ≥ 98%, Sigma Aldrich), ethanol (C_2_H_5_OH, HPLC grade, Fisher Scientific), methanol (CH_3_OH, HPLC grade, Fisher Scientific), deionized water (18.2 MΩ) was collected from an ELGA PURELAB flex apparatus.

### Synthesis of copper dimer complex

Solutions A, B, and C were prepared by ultrasonically dispersion method, respectively. The detailed preparation process was as follows: Solution A: 1.6 mmol 272 mg CuCl_2_·2H_2_O was ultrasonically dispersed in 20 mL deionized water; Solution B: 1.6 mmol 248 mg 2,2,-bipyridine was ultrasonically dispersed in 10 mL methanol; Solution C: 0.8 mmol 100 mg oxalic acid was ultrasonically dispersed in 10 mL deionized water; Subsequently, Adding solution B and solution C to solution A drop by drop respectively and kept stirring for 1 h. Finally, the light-blue solid was obtained by centrifugation, washing with water and methanol for three times and drying in vacuum^29^.

### Synthesis of g-C_3_N_4_

20 g of urea was placed to an alumina crucible (100 mL). Subsequently, the crucible was sealed with multiple layers of tin foil and put into a muffle furnace with the heating program from 50 °C to 550 °C for 2 h at the rate of 20 °C min^−1^. The obtained powder was further repeated the above calcination operation, the difference was that the heating rate was kept at 5 °C min^−1^ and the retention time at 550 °C was 3 h. Finally, the yellowish-white powder was obtained.

### Synthesis of Cu_2_@C_3_N_4_

Solution A: 0.5 g g-C_3_N_4_ was ultrasonically dispersed in 50 mL methanol solution; Solution B: 42 mg copper dimer was ultrasonically dispersed in 5 mL methanol solution; Solution B was drop-wisely added to solution A and was stirred at room temperature for 24 h, and the obtained solid was calcined in muffle furnace with the heating program from 50 °C to 250 °C for 10 h at the rate of 2 °C min^−1^. Finally, the blue-yellow solid was got.

### Synthesis of Cu_1_@g-C_3_N_4_

^64^ 3 g dicyandiamide and 340 mg CuCl_2_·2H_2_O were grounded to be-well mixed, then spread in an alumina crucible (100 mL) with a cap covered. The crucibles were places in a muffle furnace, and gradually heated to 550 °C for 8 h with the ramping rate of 5 °C min^−1^ and then cool down.

### Material characterization

X-Ray Diffraction (XRD) patterns were obtained from a PANalytical X’Pert3 X-ray diffractometer equipped with a Cu K*α* radiation source (*λ* = 1.5406 Å). Nitrogen adsorption measurements were measured on a Micromeritics ASAP 2010 instrument with the samples degassed under vacuum at 300 °C for 4 h. Specific surface area (SSA) was calculated using the Brunauer-Emmett-Teller (BET) theory. The Cu contents were determined by inductively coupled plasma mass spectrometry (ICP-MS) using a PerkinElmer Elan DRC II Quadrupole ICP-MS after dissolution of the samples in aqua regia. High angle annular dark field (HAADF) STEM images were acquired using a JEOL TEM/STEM ARM 200CF (equipped with an Oxford X-max 100TLE windowless X-ray detector) at a 22 mrad probe convergence angle and a 90 mrad inner-detector angle. The analysis of surface elements was performed on X-ray photoelectron spectroscopy (XPS), Thermo Fisher Scientific Escalab 250Xi spectrometer with Al Kα radiation as the excitation source. Fourier Transform Infrared Spectroscopy were carried out on ThermoNicolet Nexus 670. Diffuse reflectance ultraviolet-visible (UV-Vis) spectra were collected on a Shimadzu UV-2450 spectrometer equipped with an integrating sphere attachment using BaSO_4_ as the reference. FTIR Spectrometer Ultraviolet photoelectron spectroscopy (UPS) measurements were performed on an ESCALAB 250 UPS instrument with a He Iα gas discharge lamp operating at 21.22 eV and a total instrumental energy resolution of 90–120 meV.

XAS experiments were performed at the 10-BM beamline at the Advanced Photon Source (APS) at Argonne National Laboratory. Samples were pressed into a stainless-steel sample holder. All measurements were performed at the Cu K edge (8.9789 keV) in transmission mode in fast scan from 250 eV below the edge to 800 eV above the edge. Spectra processing, including background removal and normalization were performed on ATHENA module in Demeter package. The extraction of structural parameters and fitting of the DFT optimized models of fresh and spent Cu_2_@C_3_N_4_ samples were performed on ARTEMIS module. For the optimized structure, EXAFS data were fit from *k* = 2.7 to 10 Å^−1^ (dk = 2) and *R* = 1–3.2 Å with a Hanning window.

Electron Paramagnetic Resonance (EPR) measurements were performed on a Bruker EMX EPR spectrometer at X-band frequency (9.46 GHz). 5,5’-Dimethyl-1-pyrroline-N-oxide (DMPO) was used as the spin-trapping agent, which can capture the radicals •CH_3_, •OOH and •OH. As for the detection of •OOH and •OH, methanol and DI H_2_O were used respectively, due to the DMPO-OOH is not stable in H_2_O, would be quickly converted to DMPO-OH.

The in-situ irradiation X-Ray photoelectron spectroscopy (ISI-XPS) was carried out on AXIS SUPRA (Kratos Analytical Inc, Shimadazu) coupled with a continuous tunable wavelength light optical fiber (PLS-EM 150, Beijing Perfectlight Co. Ltd.). The wavelength of irradiation light was set at 400–500 nm to mimicking the visible light. The measurement setup is developed to monitor the photoelectron transfer process. Before measurement, the hydrated Cu_1_@g-C_3_N_4_ was obtained by pretreatment of fresh Cu_1_@g-C_3_N_4_ by water.

## Supplementary information

Supplementary Information

Peer review file

## Data Availability

The authors declare that the data supporting the findings of this study are available within the paper and its Supplementary Information file. The data generated in this study for main manuscript are provided in the Source Data file. Other raw data of the presented figures and tables are available from the corresponding authors upon request. Source data are provided with this paper.

## Source data

Source Data
