# Supplementary material for: Oxo dicopper anchored on carbon nitride for selective oxidation of methane
Source: Nat Commun. 2022 Mar 16;13:1375. doi: 10.1038/s41467-022-28987-1 (PMC8927601; doi:10.1038/s41467-022-28987-1)
Supplement: Supplementary file 1 — Supplementary Information [file 41467_2022_28987_MOESM1_ESM.pdf]

Supplementary Materials

Oxo Dicopper Anchored on Carbon Nitride for Selective Oxidation of Methane

*Pengfei Xie,[1, 2 †,] * Jing Ding,[1, 3 †] Zihao Yao,[4 †] Tiancheng Pu,[1 †] Peng Zhang,[5] Zhennan Huang,[6] Canhui Wang,[1] Junlei Zhang,[1] Noah Zecher-Freeman,[1] Han Zong,[1] Dashui Yuan,[3] Shengwei Deng,[4] Reza Shahbazian-Yassar,[6] Chao Wang[1,]**

[1] Department of Chemical and Biomolecular Engineering, Johns Hopkins University, Baltimore, Maryland 21218, United States

[2] College of Chemical and Biological Engineering, Zhejiang University, Hangzhou 310027, China

[3] State Key Laboratory of Materials-Oriented Chemical Engineering, College of Chemical Engineering, Nanjing Tech University, Nanjing 210009, P.R. China

[4] Institute of Industrial Catalysis, College of Chemical Engineering, Zhejiang University of Technology, Hangzhou 310032, China

[5] School of Materials Science and Engineering, Zhengzhou University, Zhengzhou 450001, China

[6] Department of Mechanical and Industrial Engineering, University of Illinois, Chicago, Illinois 60607, United States

KEYWORDS: *copper dimer catalysts, dicopper-oxo centers, selective oxidation of methane, heterogeneous catalysis, photocatalysis*

† These authors contributed equally

* Corresponding Email: chaowang@jhu.edu; pfxie@zju.edu.cn

SUPPLEMENTAL METHODS

Thermo-catalytic measurements. The selective methane oxidation was performed in a high-pressure Parr reactor. 0.2 mmol H_2O_2 dissolved in 10 ml deionized H_2O was used as the oxidizing agent. 50 mg of catalyst powder was added to the aqueous solution. After evacuating the air left in reactor by flowing methane (0.1 MPa) and purging for five times, the system then was pressurized with argon to 3 MPa. The solution was vigorously stirred at 1500 rpm, meanwhile heated to 50 ^{o}C. Both temperature and pressure were well controlled and kept constant during catalysis. The reaction time of all experiments was strictly controlled at certain time (e.g., 30 mins, 1 or 2 h) after the temperature of solution reaches a pre-set temperature. After the reaction, the reactor was set in an ice bath to cool down immediately, the solution was kept being stirred at 1500 rpm.

Upon the reaction completely cooled down to ice bath temperature, the gas components (i.e., CH_4, CO_2) were injected and determined with gas chromatograph equipped with a BID detector (GC-2010 plus, Shimadzu). Before analysis, we used the gas in autoclave to sweep the GC lines for 20 s. The solution consisting of liquid products was filtered from catalyst powder. The liquid products including CH_3OOH and CH_3OH and others was quantitatively analyzed with ^{1}H-NMR. Typically, 0.7 mL of sample and 0.1 mL of D_2O were placed in an NMR tube along with 4,4-dimethyl-4-silapentane-1-sulfonic acid (DSS, δ=0 ppm) as the internal standard. During NMR measurements, a solvent suppression program was run in order to minimize the signal

originating from H_2O. A typical ^1H-NMR spectrum is provided in Supplementary Figure 8. The identified oxygenated products were methanol (δ=3.34 ppm), methyl hydroperoxide (δ=3.85 ppm). Ratios of peak areas of methanol or methyl peroxide to peak area of DSS were calculated. Then the products are determined by using the standard curves of methanol, methyl hydroperoxide built in Supplementary Figure 9-S10.

The H_2O_2 concentration was measured by a traditional cerium sulfate $Ce(SO_4)_2$ titration method based on the mechanism that a yellow solution of Ce^{4+} would be reduced by H_2O_2 to colorless Ce^{3+} ($2Ce^{4+} + H_2O_2 \rightarrow 2Ce^{3+} + 2H^+ + O_2$). Thus, the concentration of Ce^{4+} before and after the reaction can be measured by ultraviolet-visible spectroscopy. The wavelength used for the measurement was 316 nm. The standard curve of H_2O_2 was built in Supplementary Figure 18.

Photo-catalytic studies. The photocatalytic methane oxidation reaction tests were conducted in a 50 mL batch-reactor equipped with a quartz window to allow light irradiation. Typically, 50 mg catalyst was dispersed in 10 mL deionized water by ultrasonication for 10 min. Then the mixture was added into the reaction cell, and the reaction cell was placed in the batch-reactor. The batch-reactor was purged with 0.1 MPa CH_4 and 0.1 MPa O_2 for five times to exhaust air, then the reactor was pressurized with argon to 3 MPa. To study the influence of different CH_4 or O_2 partial pressure on the photocatalytic reaction, 0.5, 1 MPa CH_4 with 0.1 MPa O_2 or 0.1 MPa CH_4 with 0.5 MPa O_2 were also applied. Subsequently, the reactor was stirred at 50 ℃ under the light irradiation provided by a 300 W xenon lamp (MC-XS500, Testmart), equipped with a 420 nm optical filter (Ceaulight), the light intensity was controlled at 100

mW/cm. A thermocouple was inserted into the solution to directly detect the temperature of the liquid solution. During the process, the temperature was maintained at 50 ℃. After the reaction, the reactor was cooled in an ice bath to a temperature below 10 ℃. The analysis of products followed the same protocol as shown above. The conversion of CH_4, the selectivity of products, and the mass reaction rate were calculated according to the following equations:

$$\mathrm{mol}_{\mathrm{CH4,\ input}} = \frac{\mathrm{mol}_{\mathrm{Ar}}}{\text{GC Peak area of Ar}} \times f(\text{correction factor}) \times \text{GC Peak area of } \mathrm{CH_4} \quad (1)$$

$$\text{Conversion} = \frac{\mathrm{mol(CH_3OOH)+mol(CH_3OH)+mol(CO_2)}}{\mathrm{mol(CH_{4,\ input})}} \times 100\% \quad (2)$$

$$\text{Selectivity} = \frac{\text{mol(specific product)}}{\mathrm{mol(CH_3OOH)+mol(CH_3OH)+mol(CO_2)}} \times 100\% \quad (3)$$

$$\text{Productivity (mmol g}^{-1}\text{h}^{-1}) = \frac{\text{mol(specific product)}}{\text{weight of Cu (g)} \times \text{reaction time(h)}} \times 100\% \quad (4)$$

Computational Methods. In this work, all the simulations were carried out for the direct synthesis of CH_3OOH and CH_3OH within the framework of the spin-polarized generalized gradient approximation with the Perdew-Burke-Ernzerh of functional in the VASP code.[1] The cutoff energy of plane-wave basis expansion was set to 400 eV. The approach of project-augmented-wave (PAW)[2] was exploited to describe the interaction between core-electron and valence electron. The Methfessel-Paxton-approach with a fermi smearing width of 0.1 eV was used to determine partial occupancies on electronic states. Electronic convergence was set to 10^{-5} eV, and geometries were converged to less than 0.05 eV/Å. All the possible surfaces were constructed with 2 × 2 × 1 Monkhorst−Pack k-point mesh sampling which is well tested.[3] The effect of vdW interaction is significant for the reaction mechanism in our precious study.[4, 5]

Therefore, the DFT-D3 method of Grimme et al.[6] was utilized to calculate all the energetics and structures of the intermediates and transition states. All the surfaces were relaxed with a 15 Å vacuum region. The transition states (TSs) were searched using the method called a constrained optimization scheme.[7, 8] The TSs were confirmed by two rules: (i) all forces on atoms vanish; (ii) the total energy is a maximum along the reaction coordinate but a minimum with respect to the rest of the degrees of freedom. Vibrational frequency analyses were performed to confirm the integrity of TSs.[9]

SUPPLEMENTAL DATA

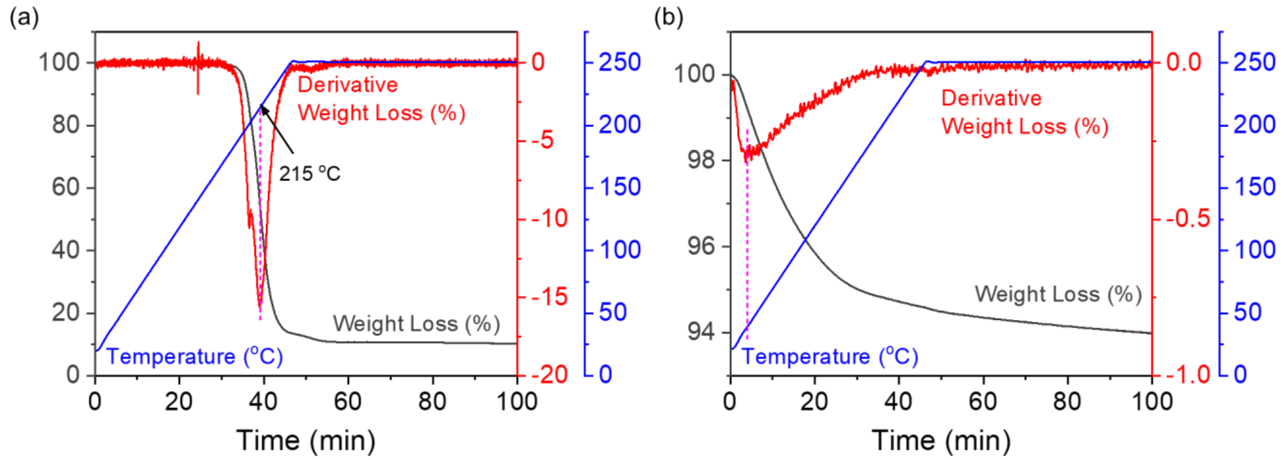

Supplementary Figure 1. Thermogravimetric analysis (TGA) profiles showing the weight loss and the corresponding first derivative values of the dimeric copper complex (a) and the pristine $Cu_2@C_3N_4$ with ligands (b) when burned in air.

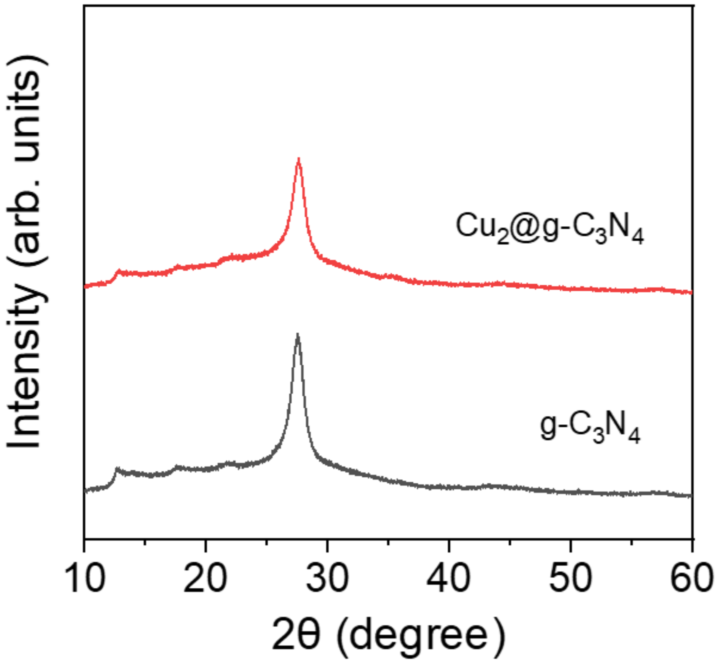

Supplementary Figure 2. XRD patterns of Cu_2@C_3N_4 and g-C_3N_4.

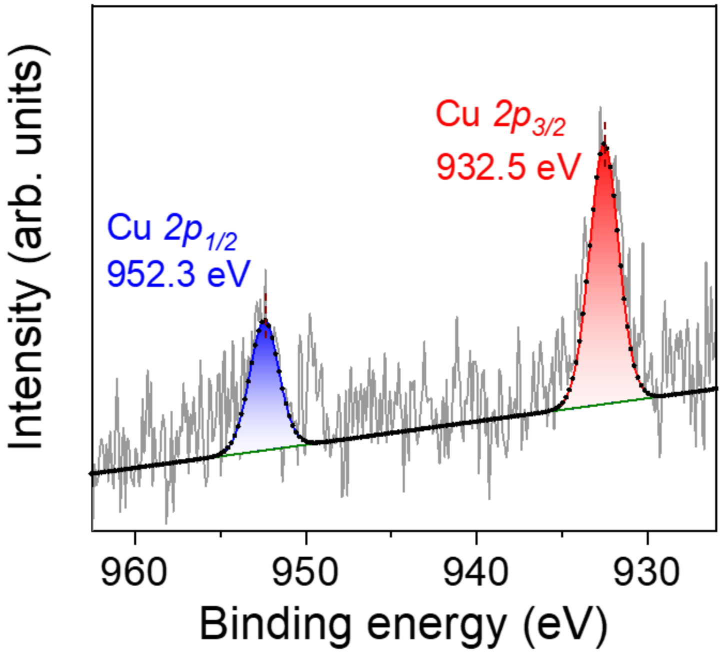

Supplementary Figure 3. XPS spectrum of Cu *2p* edge for $Cu_2@C_3N_4$

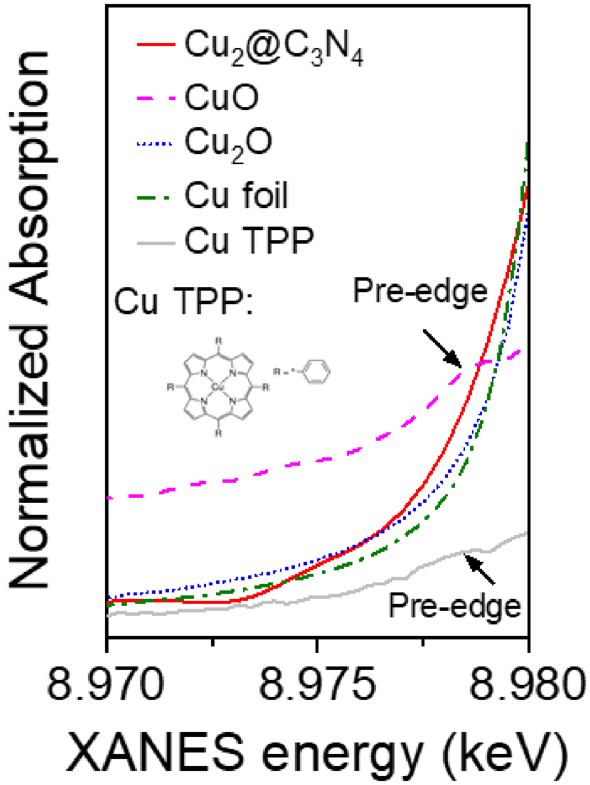

Supplementary Figure 4. Cu K-edge XANES spectra of $Cu_2@C_3N_4$, Cu foil, Cu_2O, CuO and copper tetraphenylporphyrin (Cu-TPP). Inset: the molecular structure of Cu-TPP which involves one Cu coordinated to four N via Cu-N bonding.

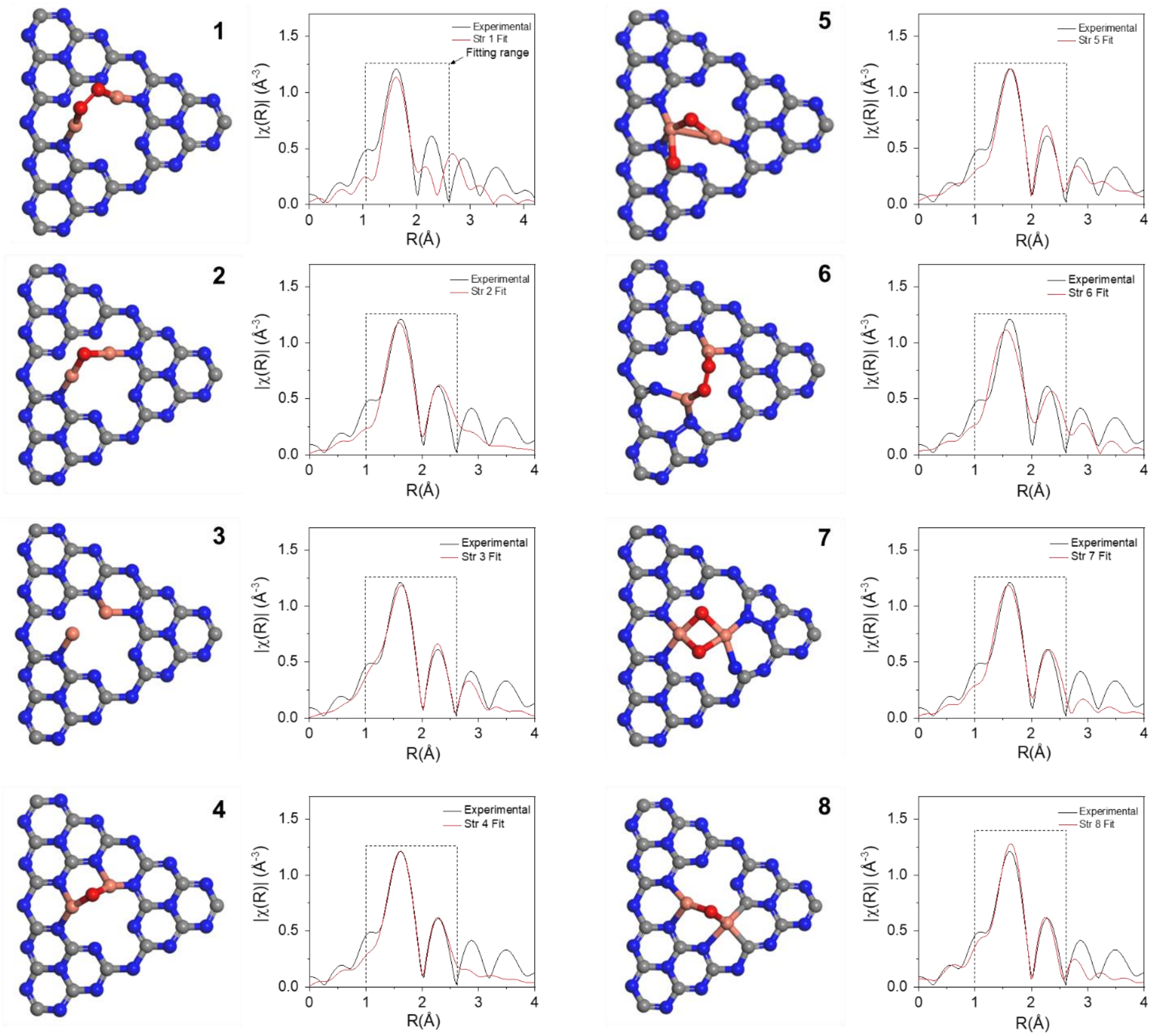

Supplementary Figure 5. Alternative Cu-dimer configurations simulated by using DFT and comparison of the corresponding EXAFS fitting to experimental spectra.

Table S1. The formation energies of different structures studied for the optimization of Cu dimer.

Structures	ΔE (eV)
1	-8.09
2	-8.74
3	-5.58
4	-8.09
5	-8.97
6	2.62
7	-6.72
8	-9.03
9	-12.74

To calculate the formation energies of potential Cu dimer structures, we used the equation

$$E(\text{formation energy}) = E(\text{System}) - E(\text{Substrate}) - a\times E(\text{Cu}) - b\times 0.5\times E(\text{O}_2) \quad (5)$$

Here, E(System) represents the total free energy of the system, E(Substrate), E(Cu) and $E(O_2)$ represent the free energies of the support, Cu atoms and oxygen gas, and a and b are the numbers of Cu and O atoms involved in the considered structure.

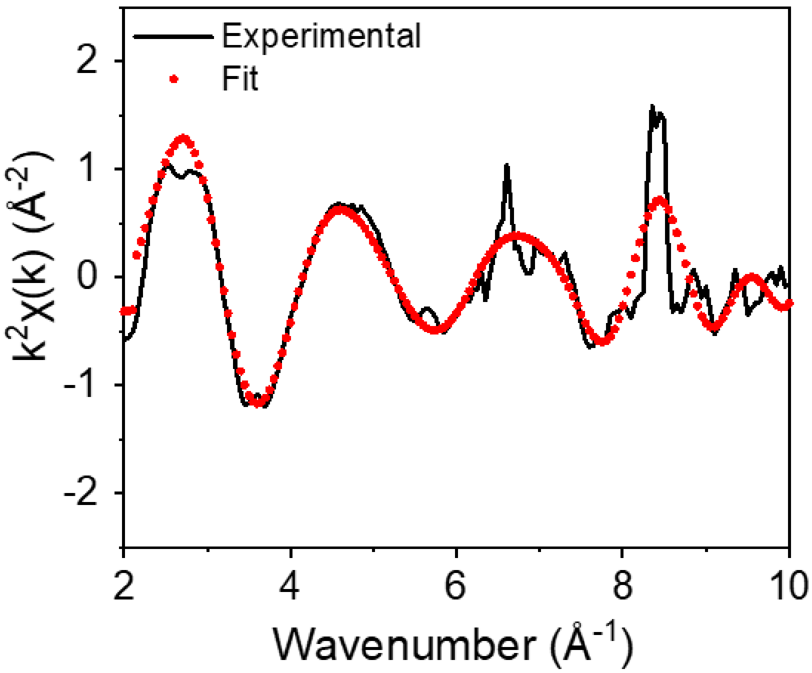

Supplementary Figure 6. The k-space fitting analysis of EXAFS spectrum for $Cu_2@C_3N_4$ with consideration of both monomeric and dimeric Cu sites. This corresponds to Figure 2d in the main text and the corresponding fitting parameters are summarized in Table 1.

(a)

Structure	Bader Charge	Oxidation state
Metallic Cu	0	0
Cu2O	0.42	+1
CuO	0.77	+2
Cu-O-Cu	0.66	+1.67

(b)

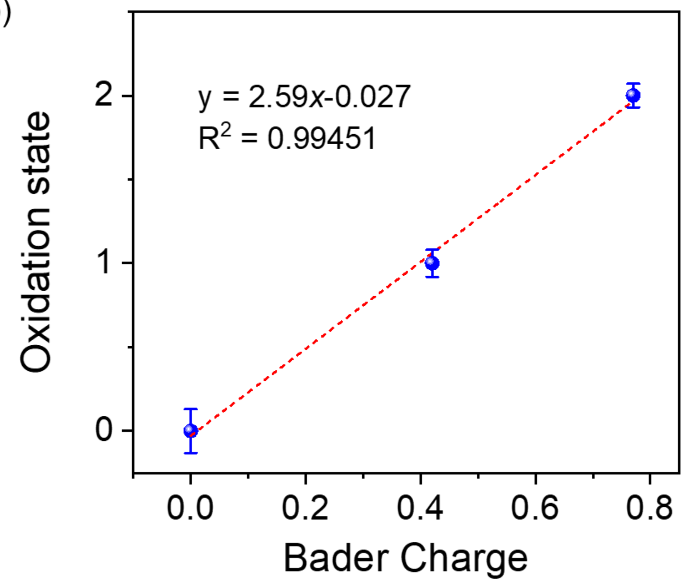

Supplementary Figure 7. Bader charge analysis for the oxidation state of Cu in $Cu_2@C_3N_4$. The error bars in b indicate the statistical distribution of the computed Cu charges. Fitting the Bader charge of Cu derived from DFT calculations into a calibration curve established based on the references of metallic Cu, Cu_2O and CuO gives an oxidation state of +1.67, which is consistence with our experimental results based on XPS and XANES.

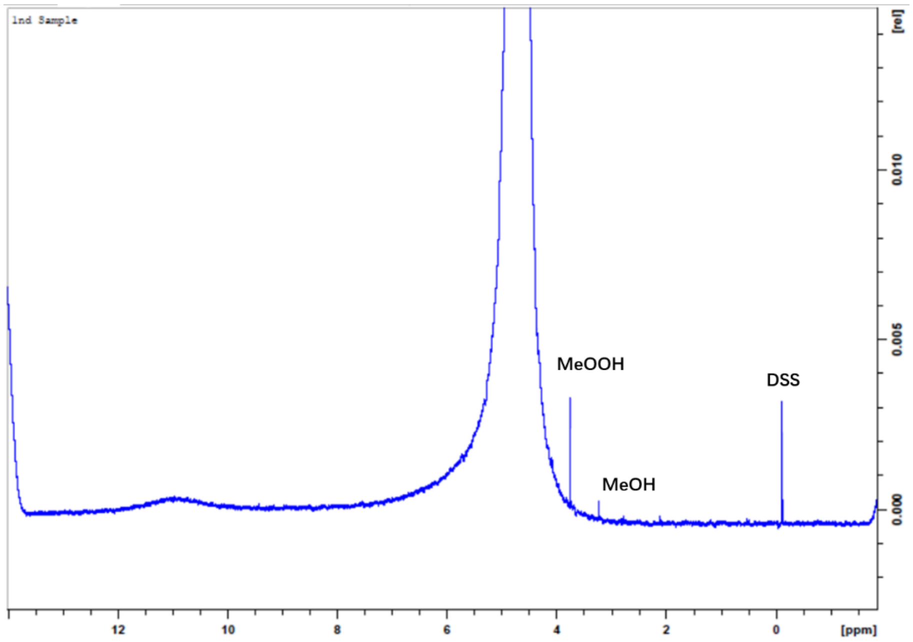

Supplementary Figure 8. A representative NMR spectrum collected for the methane oxidation products using Cu_2@C_3N_4 catalysts and H_2O_2 as the oxidizer.

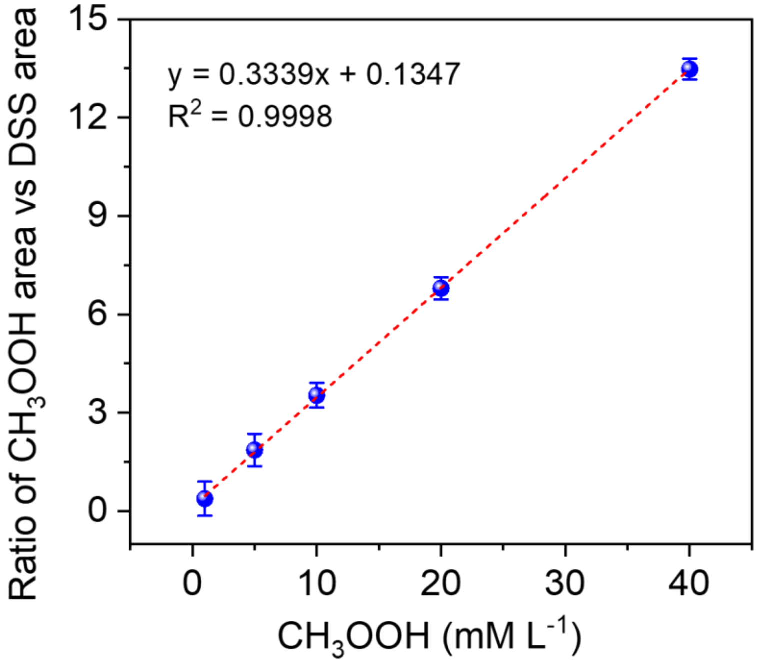

Supplementary Figure 9. Calibration curve for the analysis of CH_3OOH using NMR. The error bars indicate the statistical distribution derived from three independent measurements.

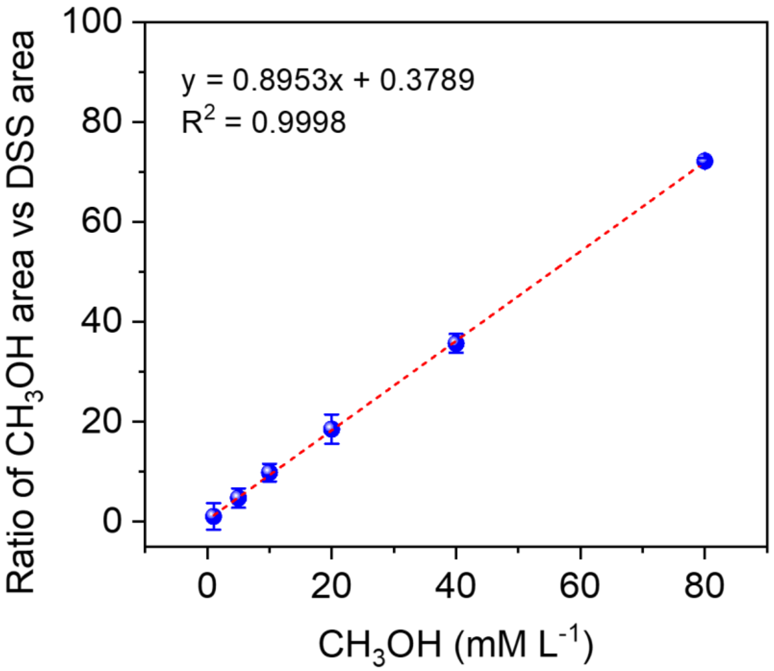

Supplementary Figure 10. Calibration curve for the analysis of CH_3OH using NMR. The error bars indicate the statistical distribution derived from three independent measurements.

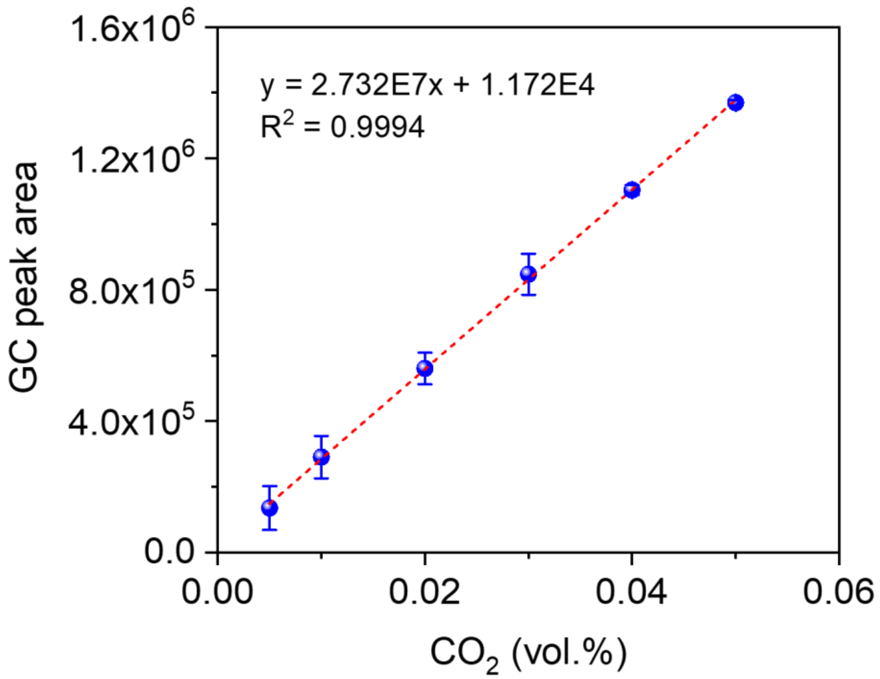

Supplementary Figure 11. Calibration curve for the GC analysis of CO_2. The error bars indicate the statistical distribution derived from three independent measurements.

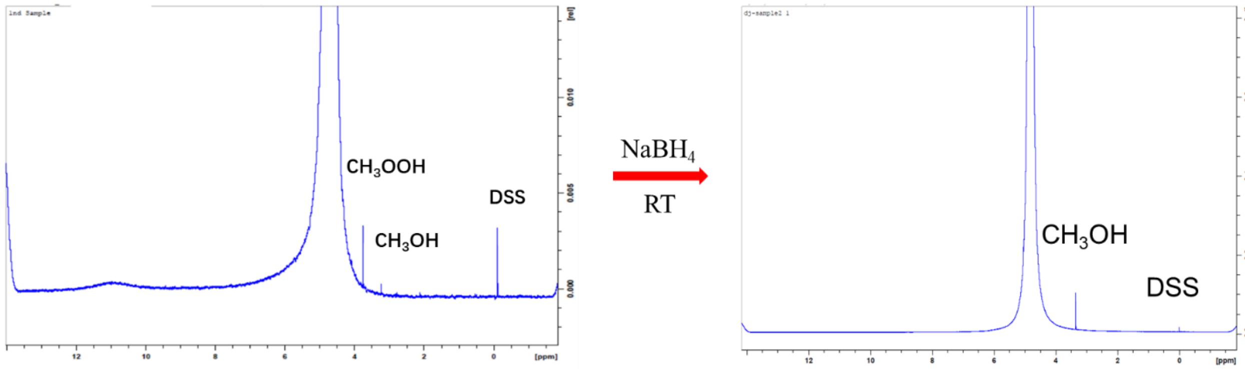

Supplementary Figure 12. Room temperature reduction of CH_3OOH with $NaBH_4$ to form CH_3OH.

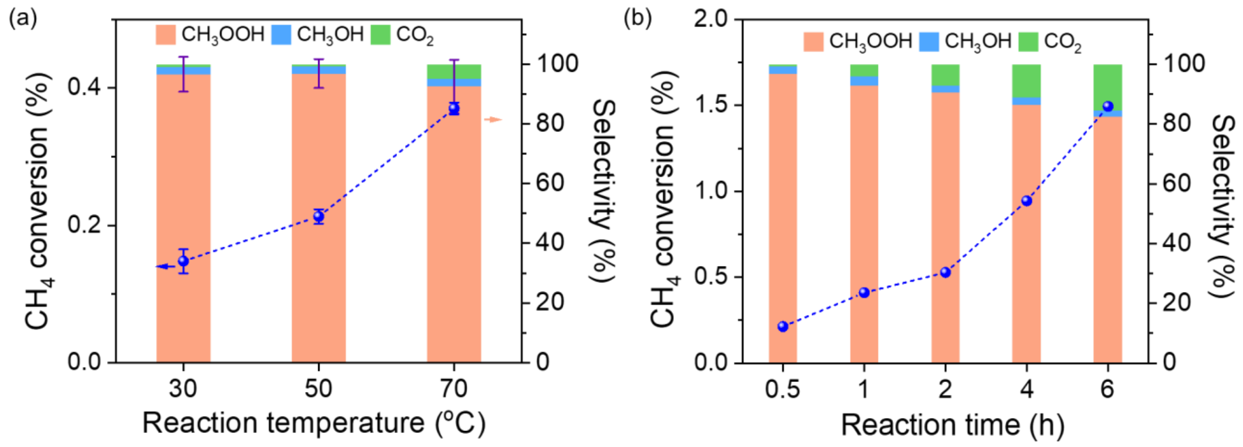

Supplementary Figure 13. CH_4 conversion and product selectivity of the thermo-catalytic oxidation of methane using H_2O_2 and $Cu_2@C_3N_4$. (a) Dependence on reaction temperature and (b) time (at 50 °C). The results indicate that evaluated temperature and prolongated reaction time lead to the overoxidation of CH_4 to CO_2, which is the thermodynamically most stable product. The error bars indicate the statistical distribution derived from three independent measurements.

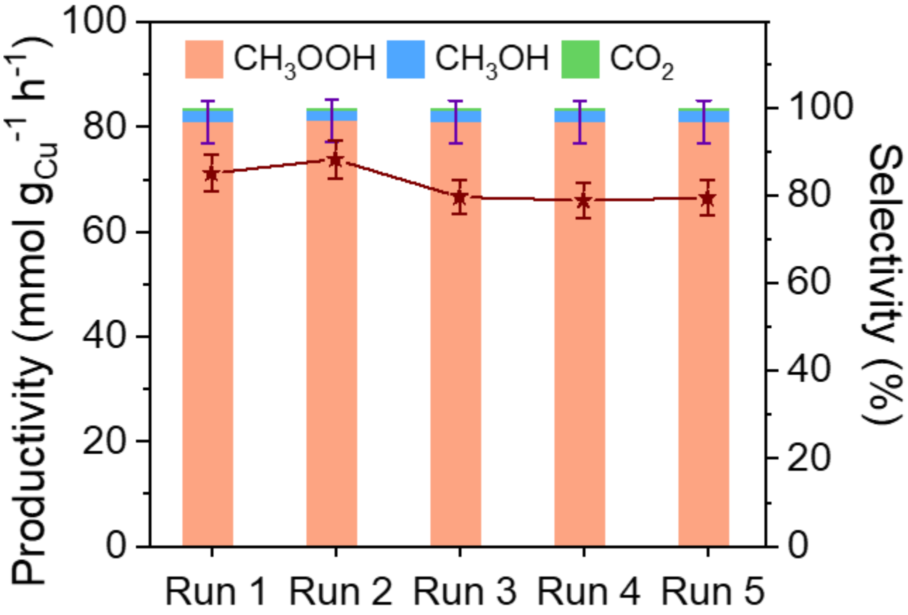

Supplementary Figure 14. Cycling test of $Cu_2@C_3N_4$ for thermo-catalytic oxidation of CH_4 with H_2O_2. The error bars indicate the statistical distribution derived from three independent measurements.

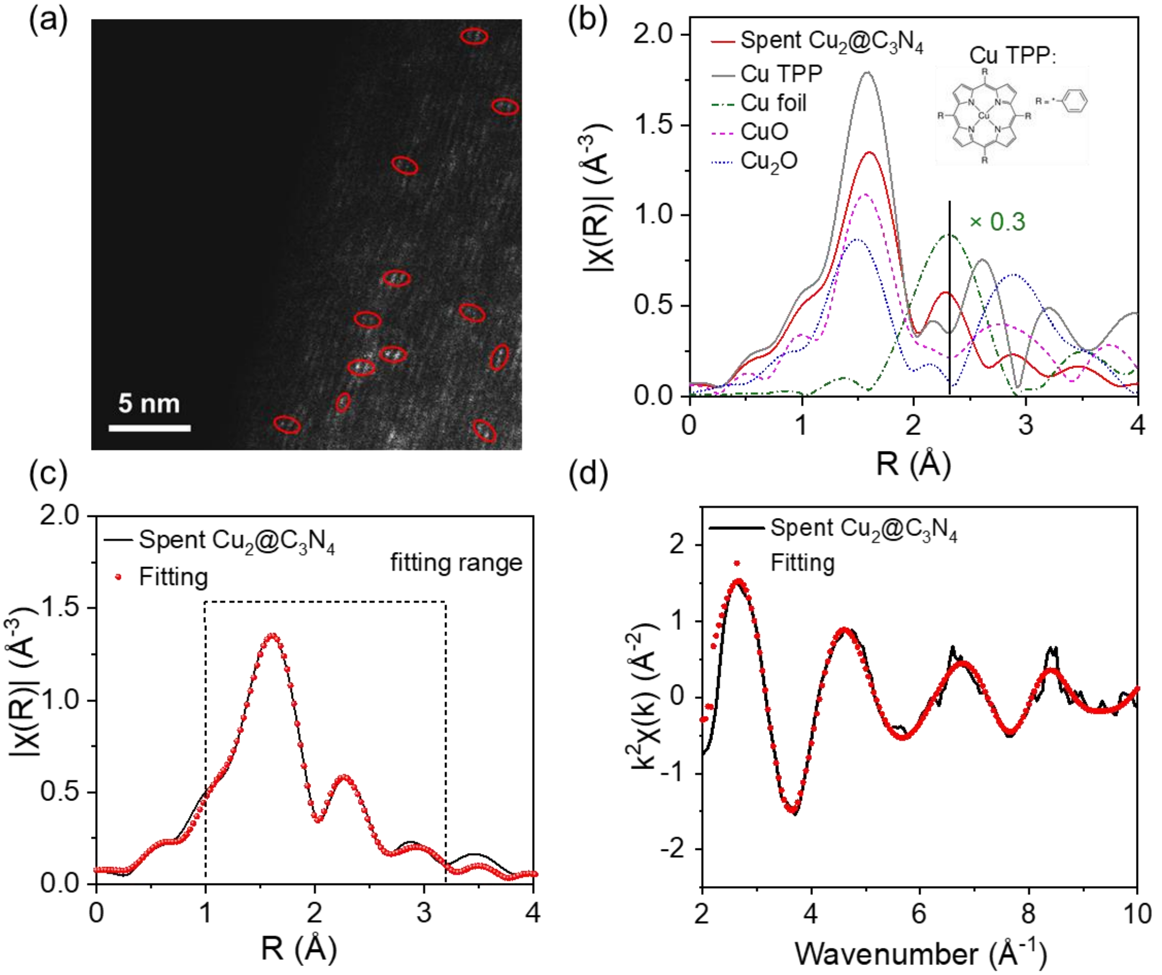

Supplementary Figure 15. Characterizations of the spent $Cu_2@C_3N_4$ catalyst after the 6-h durability test. (a) Representative HAADF-STEM images with Cu dimers highlighted using red circles. (b) k^2-weighted EXAFS spectra at the Cu K edge, with Cu foil, Cu_2O, CuO and Cu-TPP (one Cu coordinated with for N atoms) being used as the references. (c, d) Fitting of the EXAFS spectrum with consideration of both monomeric and dimeric Cu sites.

Table S2. Structural parameters being used to fit the EXAFS spectrum of the spent $Cu_2@C_3N_4$ catalyst after 6 h of reaction.

**Scattering path**	**CN**	**Distance (Å)**	**σ^2(Å^2)**	**R-factor**
Cu-O	1.35±0.24	1.77±0.01	0.0049±0.0005	0.008±0.001
Cu-N (dimer)	1.91±0.33	1.99±0.01		
Cu-N (single atom)	2.42±0.32	1.98±0.01		
Cu-Cu	0.81±0.14	2.71±0.01	0.0043±0.0004	

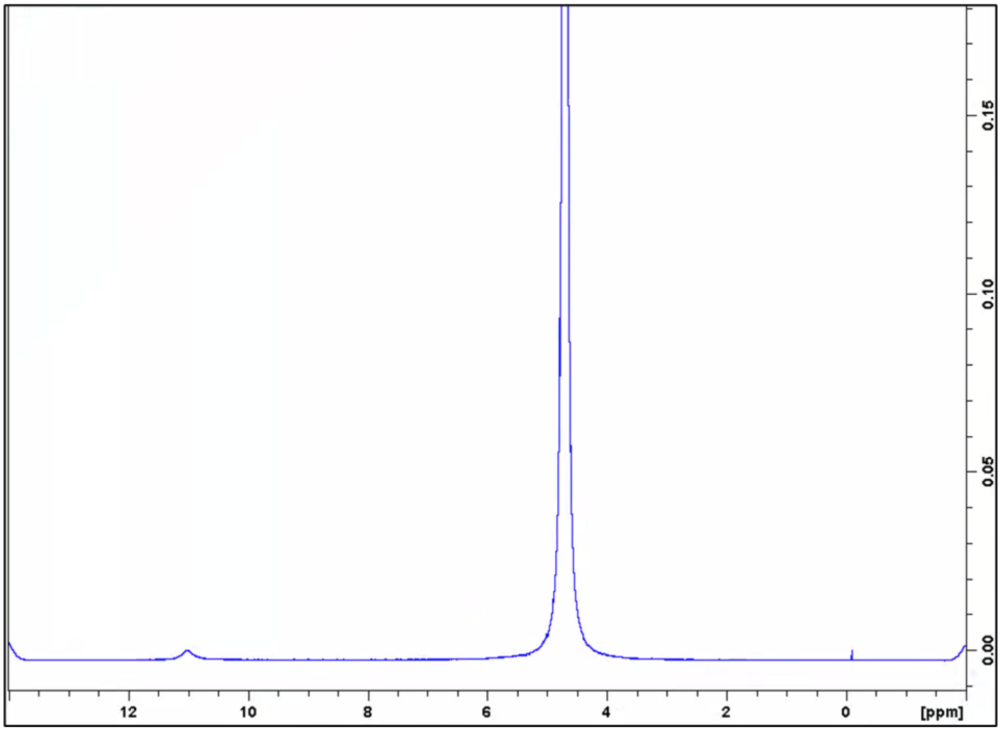

Supplementary Figure 16. NMR spectrum collected for the control experiment using bare g-C_3N_4. No oxidation product was detected.

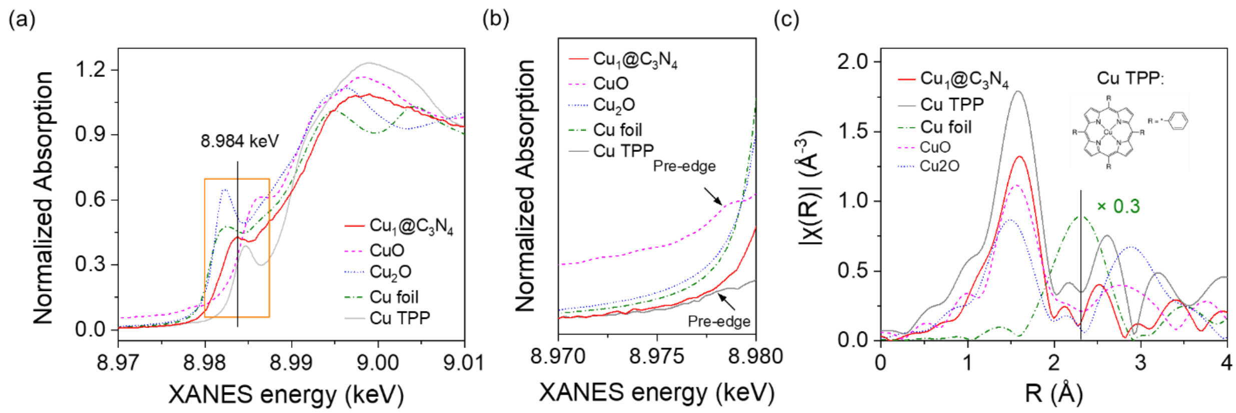

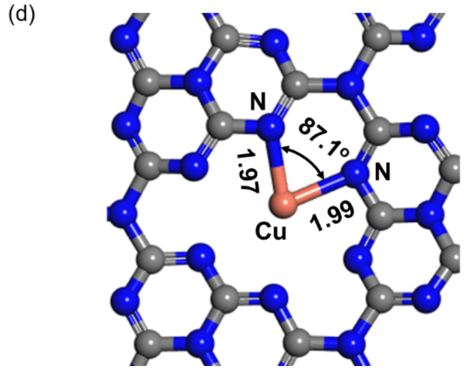

(e)

Scattering path	Coordination number	Average distance (Å)	σ^2(Å^2)	R-factor
Cu-N	2.42±0.32	1.98±0.01	0.0045±0.0006	0.008±0.002

Supplementary Figure 17. (a, b) XANES and (c) EXAFS spectra collected at the Cu K edge for $Cu_1@C_3N_4$. Cu foil, Cu_2O, CuO and Cu-TPP (1 Cu coordinated with 4 N atoms) were also shown as references. (d) The optimized structure of $Cu_1@C_3N_4$ based on DFT calculations. (e) Fitting parameters for the EXAFS spectrum of $Cu_1@C_3N_4$.

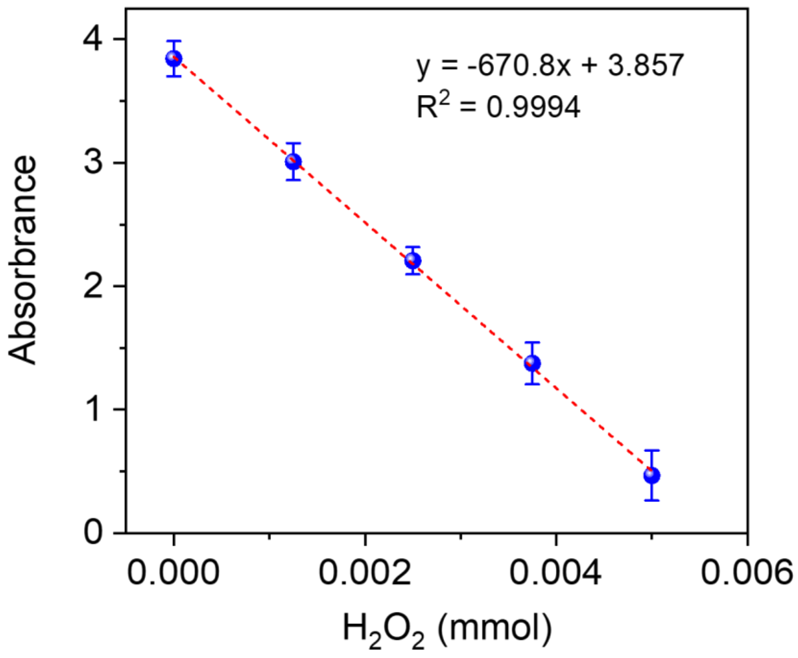

Supplementary Figure 18. The calibration curve of H_2O_2 quantified by the titration of $Ce(SO_4)_2$. The error bars indicate the statistical distribution derived from three independent experimental measurements.

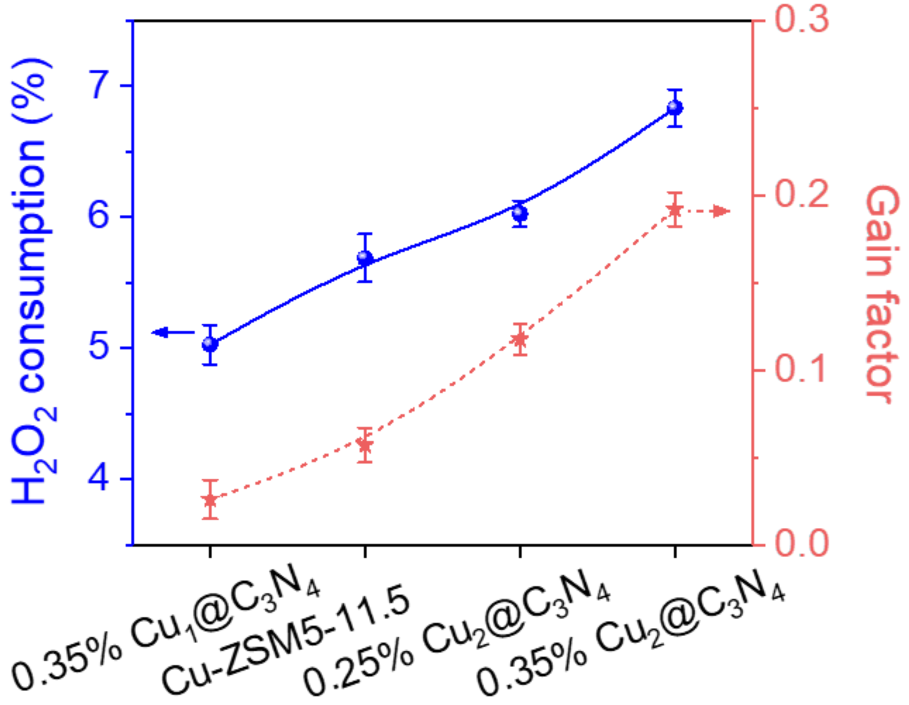

Supplementary Figure 19. Comparisons of H_2O_2 consumption and gain factors (denoted as mol of CH_3OH and CH_3OOH divided by total mol of H_2O_2 consumed) over different catalysts. The error bars indicate the statistical distribution derived from three independent experimental measurements.

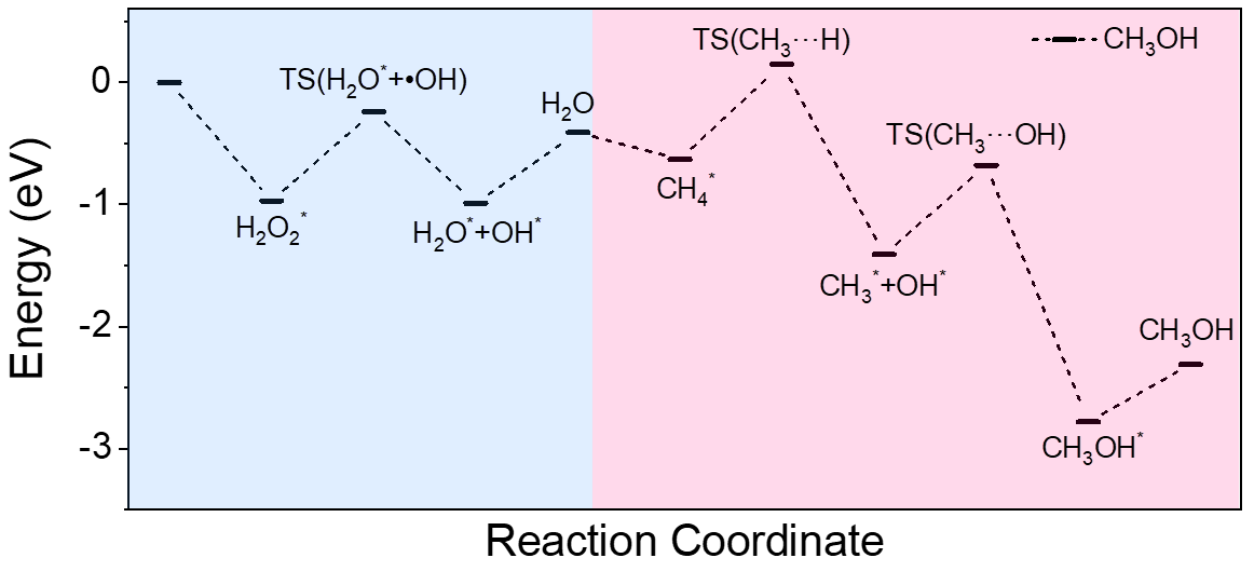

Supplementary Figure 20. Free energy diagram for the second reaction pathway of methane partial oxidation to CH_3OH on fresh $Cu_2@C_3N_4$ catalysts.

Table S3. Energy barriers and enthalpies for elementary steps during the thermal catalytic CH_4 selective oxidation by H_2O_2 to CH_3OH on fresh Cu dimer of $Cu_2@C_3N_4$.

	**Elementary steps**	E_a (eV)	ΔH (eV)
R1	H_2O_2+* → H_2O_2*	0.00	-0.97
R2	H_2O_2*+H*→OH*+H_2O*	0.73	-0.02
R3	H_2O*→H_2O+*	0.58	0.58
R4	CH_4+*→CH_4*	0.00	-0.22
R5	CH_4*→CH_3*+H*	0.76	-0.78
R6	CH_3*+OH*→CH_3OH*	0.73	-1.37
R7	CH_3OH*→CH_3OH+*	0.47	0.47

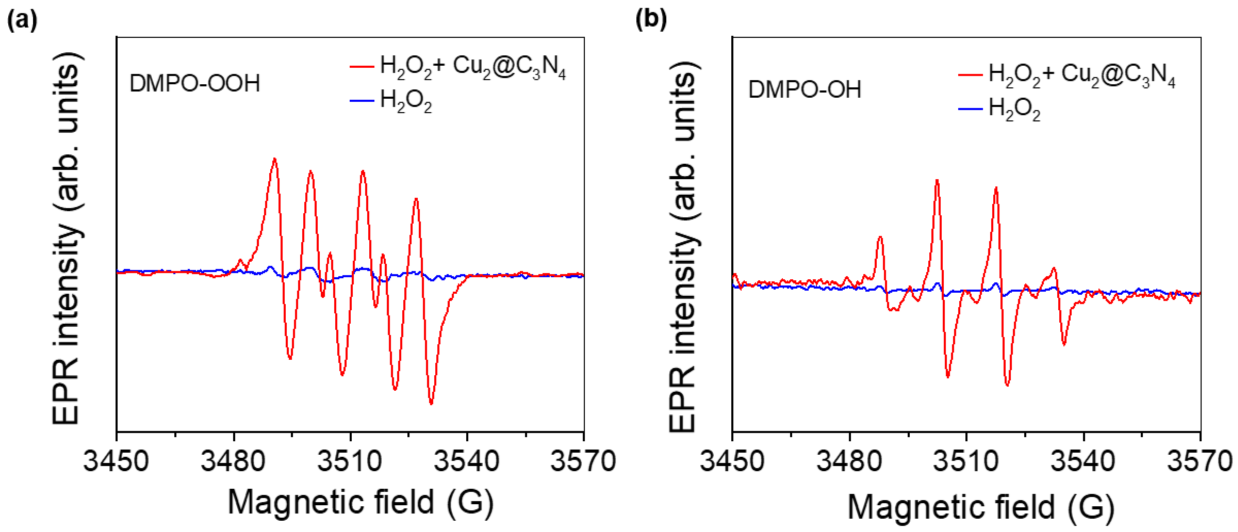

Supplementary Figure 21. EPR spectra of the radicals, •OOH in methanol (a) and •OH in H_2O (b) at different conditions with DMPO as the radical trapping agent, showing that the presence of $Cu_2@C_3N_4$ enhances the cleavage of H_2O_2 to •OOH and •OH.

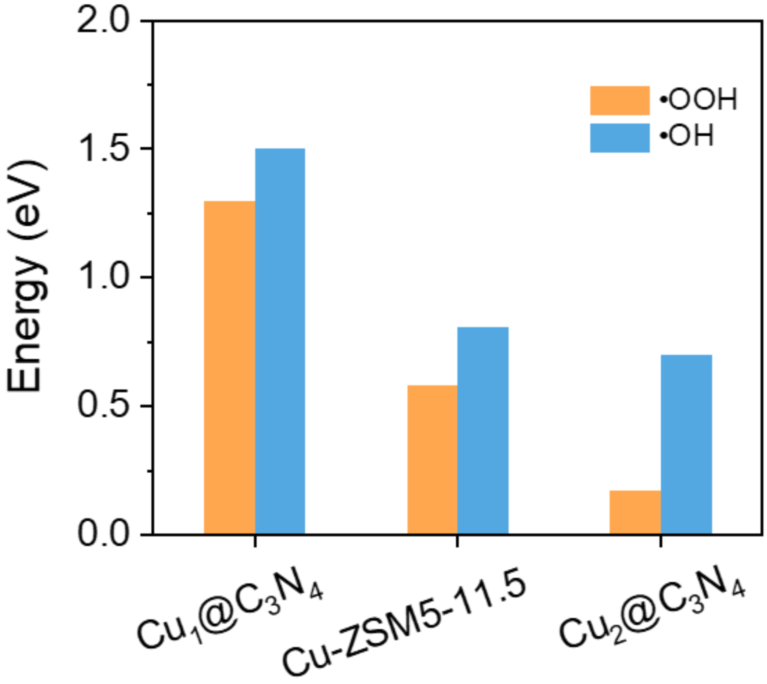

Supplementary Figure 22. Comparison of the barriers for H_2O_2 cleavage to •OOH and •OH catalyzed by different catalyst.

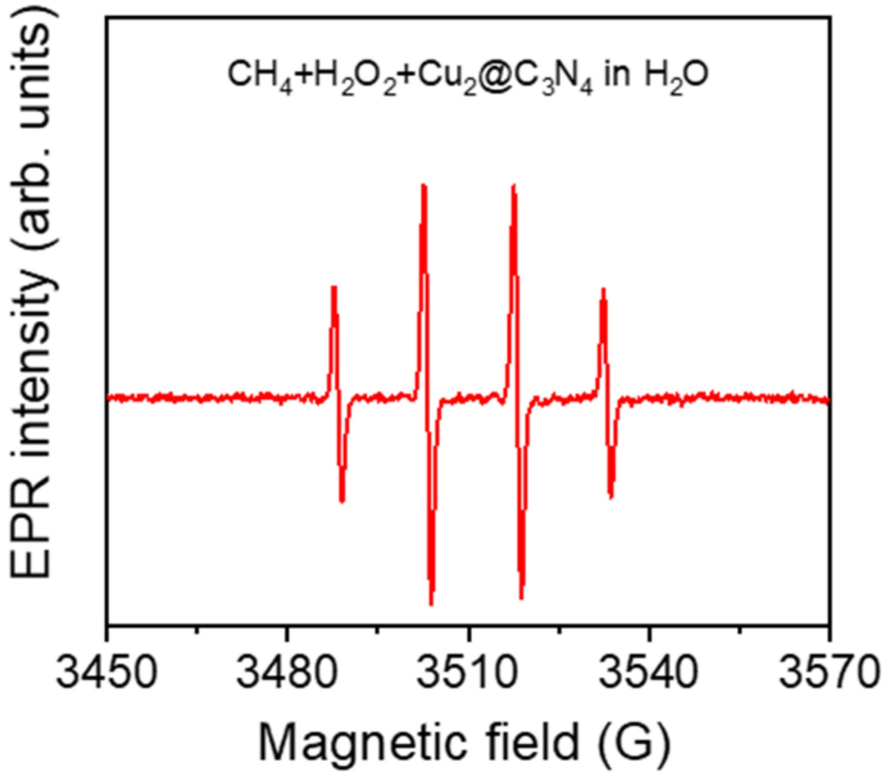

Supplementary Figure 23. In situ EPR characterization of thermal catalytic CH_4 selective oxidation by H_2O_2 on $Cu_2@C_3N_4$ with DMPO as radical trapping agent.

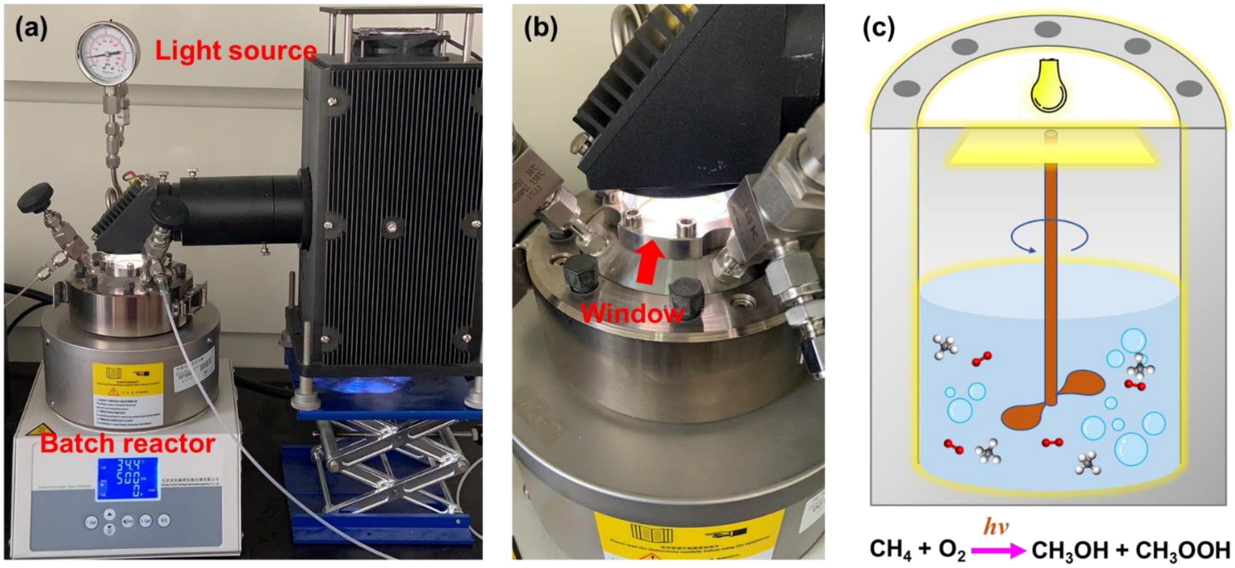

Supplementary Figure 24. (a, b) Experimental set-up of photocatalytic CH_4 oxidation by O_2. (c) Schematic illustration of the process for photocatalytic CH_4 oxidation by O_2.

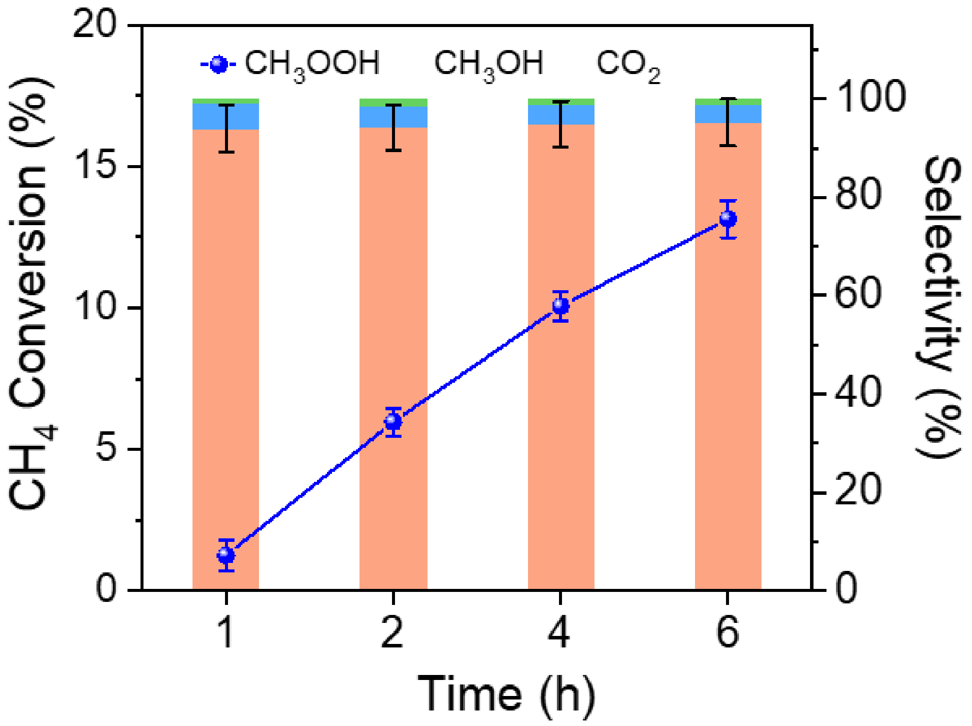

Supplementary Figure 25. The product selectivity of photocatalytic CH_4 oxidation with O_2 over $Cu_2@C_3N_4$ as a function of reaction time. The error bars indicate the statistical distribution derived from three independent experimental measurements.

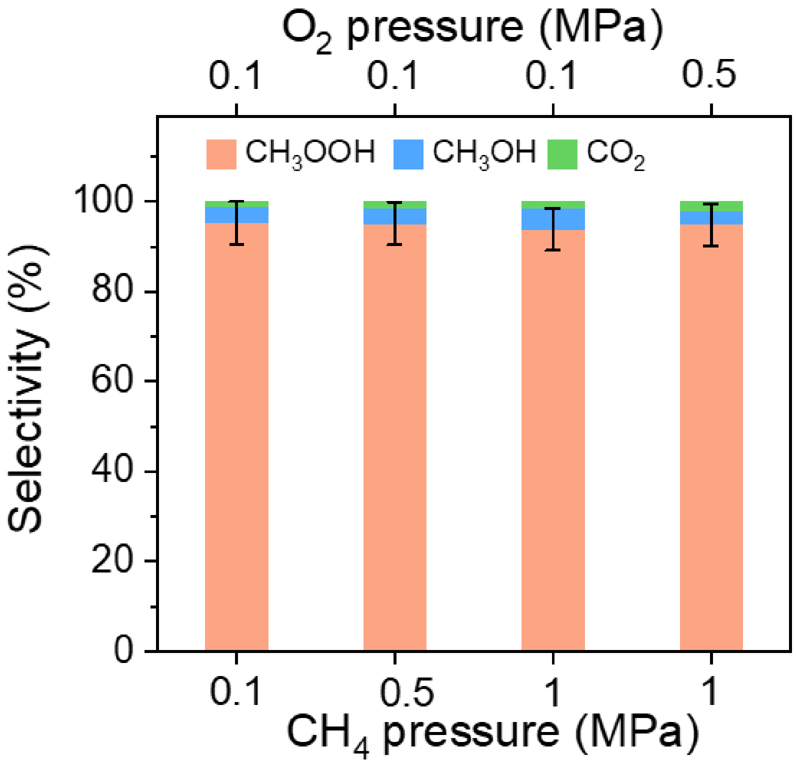

Supplementary Figure 26. The product selectivity of photocatalytic CH_4 oxidation by O_2 over $Cu_2@C_3N_4$ as function of CH_4/O_2 ratios. The error bars indicate the statistical distribution derived from three independent experimental measurements.

Table S4. Comparison of reported photocatalytic performance for partial oxidation of CH_4 with O_2.

Catalyst	Reaction conditions	CH_4 Conversion (%)	$CH_3OOH+CH_3OH$ Selectivity (%)	Productivity (mmol g_{metal}^{-1} h^{-1})	Reference
$Au_{0.75}/ZnO$	30 ^oC/2 MPa/1.5 MPa CH_4/0.5 MPa O_2	0.05	99.1	91.4	10
Au_1/Black P	90 ^oC/3.3 MPa/3 MPa CH_4/3 MPa O_2	0.01	>99%	28.4	11
0.1% Au/ZnO	30 ^oC/2.1 MPa/2 MPa CH_4/0.1 MPa O_2	0.32	95.4	12.5	12
CuSSZ-13	270 ^oC/0.1 MPa/18 kPa CH_4/0.09 kPa O_2	0.024	60	1.8	13
Bi_2WO_6	55 ^oC/0.1 MPa/0.1 MPa CH_4/ trace O_2	1.4	27.6	0.0046	14
$Pd_{0.3}Cu_{0.7}O/C$	50 ^oC/3.5 MPa/3 MPa CH_4/0.5 MPa O_2	0.09	25%	50	15
Au-CoO_x/TiO_2	25 ^oC/2.1 MPa/2 MPa CH_4/0.1 MPa O_2	0.06	95	25.4	16
$Cu_2@C_3N_4$	50 ^oC/3 MPa/0.1 MPa CH_4/0.1 MPa O_2	13.1	98.9	184.3	This work
$Cu_2@C_3N_4$	50 ^oC/3 MPa/1 MPa CH_4/0.5 MPa O_2	10.1	98.0	1399.3	This work

Table S5. Catalytic performance of multiple control experiments for the photocatalysis of selective CH_4 oxidation by O_2.

**Reaction conditions**	**Catalytic performance**
CH_4+O_2+H_2O+hυ (no Cu_2@C_3N_4)	no activity
O_2+H_2O+hυ+Cu_2@C_3N_4 (no CH_4)	no activity
O_2+H_2O+hυ+C_3N_4 (no Cu dimer)	no activity
CH_4+O_2+H_2O+Cu_2@C_3N_4 (no hυ)	no activity
CH_4+H_2O+Cu_2@C_3N_4+hυ (no O_2)	no activity

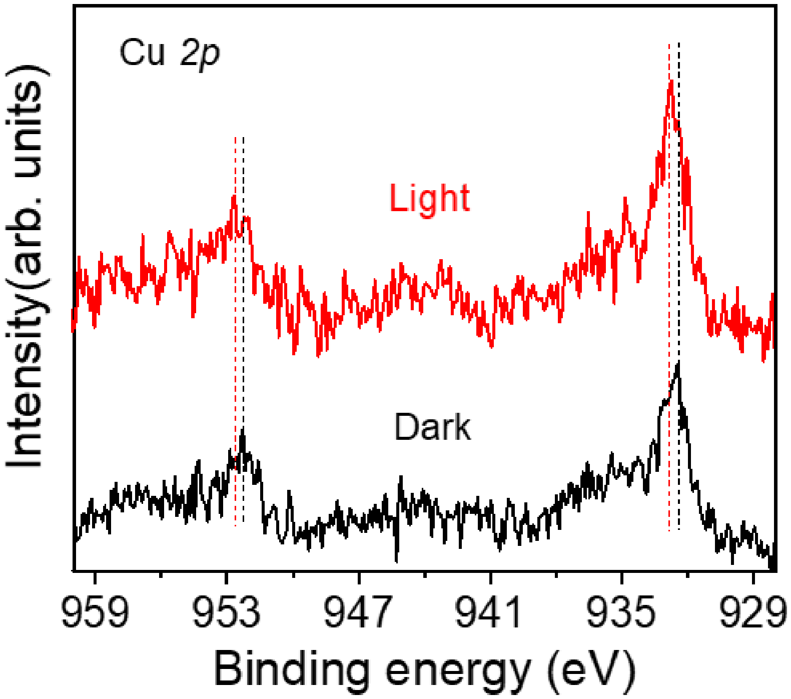

Supplementary Figure 27. *In situ* irradiation XPS characterizations of Cu *2p* edge on $Cu_2@C_3N_4$ under dark and visual light.

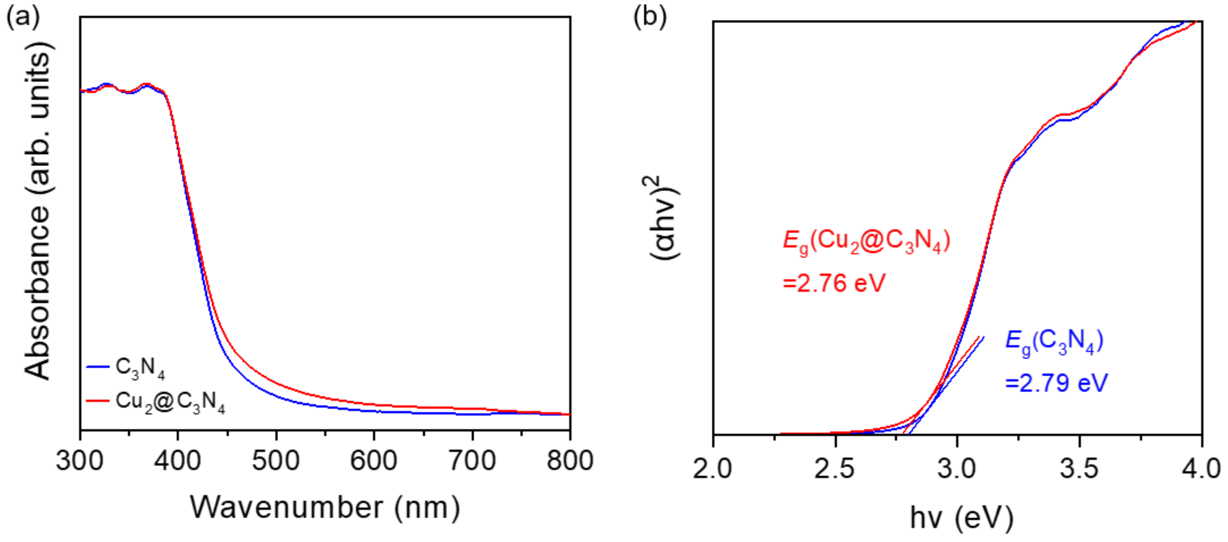

Supplementary Figure 28. (a) UV-vis DRS results of C_3N_4 and hydrated $Cu_2@C_3N_4$. (b) the corresponding Tauc plots of C_3N_4 and hydrated $Cu_2@C_3N_4$.

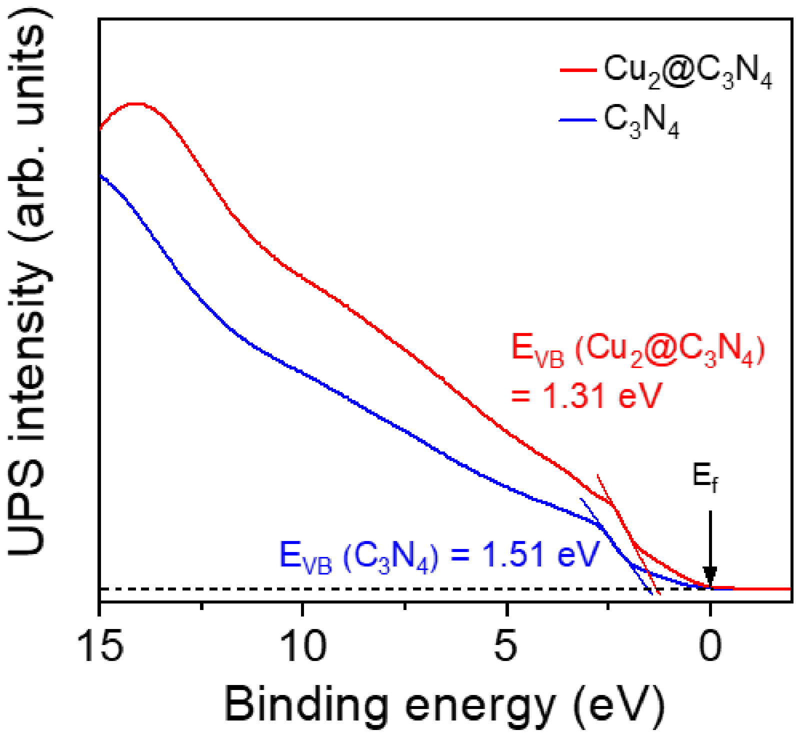

Supplementary Figure 29. UPS results of C_3N_4 and hydrated $Cu_2@C_3N_4$.

Supplementary References

1. Kresse, G. & Furthmuller, J. Efficiency of ab-initio total energy calculations for metals and semiconductors using a plane-wave basis set. *Comp. Mater. Sci.* **6**, 15-50 (1996).
2. Kresse, G. & Joubert, D. From ultrasoft pseudopotentials to the projector augmented-wave method. *Phys. Rev. B* **59**, 1758-1775 (1999).
3. Chow, H.C. & Vosko, S.H. Special Points for Two-Dimensional Brillouin-Zone or Wigner-Seitz Cell Integrations. *Can. J. Phys.* **58**, 497-503 (1980).
4. Yao, Z.H., Guo, C.X., Mao, Y. & Hu, P. Quantitative Determination of C-C Coupling Mechanisms and Detailed Analyses on the Activity and Selectivity for Fischer-Tropsch Synthesis on Co(0001): Microkinetic Modeling with Coverage Effects. *ACS Catal.* **9**, 5957-5973 (2019).
5. Wei, Z.Z. et al. Optimizing Alkyne Hydrogenation Performance of Pd on Carbon in Situ Decorated with Oxygen-Deficient TiO_2 by Integrating the Reaction and Diffusion. *ACS Catal.* **9**, 10656-10667 (2019).
6. Grimme, S., Ehrlich, S. & Goerigk, L. Effect of the Damping Function in Dispersion Corrected Density Functional Theory. *J. Comput. Chem.* **32**, 1456-1465 (2011).
7. Liu, Z.P. & Hu, P. General rules for predicting where a catalytic reaction should occur on metal surfaces: A density functional theory study of C-H and C-O bond breaking/making on flat, stepped, and kinked metal surfaces. *J. Am. Chem. Soc.* **125**, 1958-1967 (2003).
8. Zhang, C.J., Hu, P.J. & Alavi, A. A general mechanism for CO oxidation on close-packed transition metal surfaces. *J. Am. Chem. Soc.* **121**, 7931-7932 (1999).

9. Cortright, R.D. & Dumesic, J.A. Kinetics of heterogeneous catalytic reactions: Analysis of reaction schemes. *Adv. Catal.* **46**, 161-264 (2001).

10. Zhou, W.C. et al. Highly selective aerobic oxidation of methane to methanol over gold decorated zinc oxide via photocatalysis. *J. Mater. Chem. A* **8**, 13277-13284 (2020).

11. Luo, L.H. et al. Water enables mild oxidation of methane to methanol on gold single-atom catalysts. *Nat. Commun.* **12**, 1218 (2021).

12. Song, H. et al. Direct and Selective Photocatalytic Oxidation of CH_4 to Oxygenates with O_2 on Cocatalysts/ZnO at Room Temperature in Water. *J. Am. Chem. Soc.* **141**, 20507-20515 (2019).

13. Dinh, K.T. et al. Continuous Partial Oxidation of Methane to Methanol Catalyzed by Diffusion-Paired Copper Dimers in Copper-Exchanged Zeolites. *J. Am. Chem. Soc.* **141**, 11641-11650 (2019).

14. Murcia-Lopez, S., Villa, K., Andreu, T. & Morante, J.R. Partial Oxidation of Methane to Methanol Using Bismuth-Based Photocatalysts. *ACS Catal.* **4**, 3013-3019 (2014).

15. Bai, S.X., Xu, Y., Wang, P.T., Shao, Q. & Huang, X.Q. Activating and Converting CH_4 to CH_3OH via the $CuPdO_2$/CuO Nanointerface. *ACS Catal.* **9**, 6938-6944 (2019).

16. Song, H. et al. Selective Photo-oxidation of Methane to Methanol with Oxygen over Dual-Cocatalyst-Modified Titanium Dioxide. *ACS Catal.* **10**, 14318-14326 (2020).
